# Haemodynamic Predictors of Early Aortic Growth in Uncomplicated Type B Dissection: A Cohort Study

**DOI:** 10.1093/icvts/ivag198

**Published:** 2026-07-08

**Authors:** Yusuke Takei, Shohei Miyazaki, Kai Watanabe, Kohei Suzuki, Takeshi Ogasawara, Ikuko Shibasaki, Masahiro Tezuka, Shotaro Hirota, Hirotaka Ohashi, Taiki Matsuoka, Masashi Kawamura, Hirotsugu Fukuda

**Affiliations:** Department of Cardiac and Vascular Surgery, Dokkyo Medical University School of Medicine, Mibu, Tochigi 321-0293, Japan; Cardio Flow Design Inc., Chiyoda, Tokyo 102-0082, Japan; Cardio Flow Design Inc., Chiyoda, Tokyo 102-0082, Japan; Cardio Flow Design Inc., Chiyoda, Tokyo 102-0082, Japan; Department of Fundamental Education Premedical Sciences, Dokkyo Medical University, Mibu, Tochigi 321-0293, Japan; Department of Cardiovascular Surgery, Iwaki City Medical Center, Iwaki, Fukushima 973-8555, Japan; Department of Cardiac and Vascular Surgery, Dokkyo Medical University School of Medicine, Mibu, Tochigi 321-0293, Japan; Department of Cardiac and Vascular Surgery, Dokkyo Medical University School of Medicine, Mibu, Tochigi 321-0293, Japan; Department of Cardiac and Vascular Surgery, Dokkyo Medical University School of Medicine, Mibu, Tochigi 321-0293, Japan; Department of Cardiac and Vascular Surgery, Dokkyo Medical University School of Medicine, Mibu, Tochigi 321-0293, Japan; Department of Cardiac and Vascular Surgery, Dokkyo Medical University School of Medicine, Mibu, Tochigi 321-0293, Japan; Department of Cardiac and Vascular Surgery, Dokkyo Medical University School of Medicine, Mibu, Tochigi 321-0293, Japan

**Keywords:** type B aortic dissection, 4D flow MRI, haemodynamics, pre-emptive thoracic endovascular aortic repair

## Abstract

**Objectives:**

We investigated whether systolic flow-based indices derived from four-dimensional flow magnetic resonance imaging (4D flow MRI) can predict early aortic enlargement in patients with uncomplicated type B aortic dissection.

**Methods:**

This single-centre observational cohort study included 36 patients with uncomplicated type B aortic dissection between 2015 and 2025 who underwent 4D flow MRI a median of 42 days after onset with serial computed tomography (CT) follow-up. Aortic growth rates were calculated using ordinary least squares regression over the first year. False lumen (FL) flow index, FL gross flow, true lumen gross flow, and FL flow range were analysed. Correlations with aortic growth rate and group comparisons between patients with and without CT-defined aortic progression were assessed using Spearman’s rank correlation and the Mann-Whitney *U* test. Exploratory multivariable logistic regression adjusted for baseline maximum aortic diameter was also performed.

**Results:**

Sixteen patients (44%) met CT-based aortic progression criteria; 15 (42%) underwent thoracic endovascular aortic repair within 1 year. Conventional CT-derived anatomical predictors were similar between the progression and stable groups. The FL flow index (ρ = 0.44; 95% CI, 0.11-0.70) and FL gross flow (ρ = 0.46; 95% CI, 0.13-0.69) showed moderate positive correlations with aortic growth rate. On multivariable analysis, the FL flow index remained associated with progression (odds ratio, 2.31; 95% CI, 1.04-5.13) after adjustment for baseline maximum aortic diameter.

**Conclusions:**

These hypothesis-generating findings suggest that systolic FL-dominant flow may complement CT morphology in identifying patients at risk of early aortic progression.

## Graphical Abstract

**Figure:**
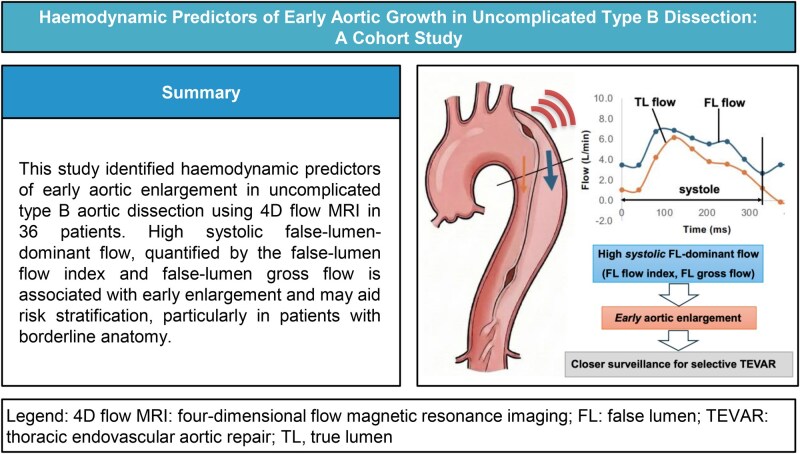

## INTRODUCTION

Management of uncomplicated type B aortic dissection (uTBAD; Stanford type B dissection without rupture or malperfusion) has undergone a paradigm shift.^1–4^ Although optimal medical therapy (OMT) remains the standard of care, pre-emptive thoracic endovascular aortic repair (TEVAR) reduces aorta-related events and promotes aortic remodelling^5^^,^^6^ without confirmed mortality benefit.^7–9^ To avoid overtreatment, current guidelines recommend interventions for patients exhibiting ‘high-risk features’,^10–12^ but accurate clinical identification of these patients is challenging.

Risk stratification relies predominantly on morphological features assessed by computed tomography (CT), including maximum aortic diameter (MAD), false lumen (FL) diameter, primary entry diameter, and partial FL thrombosis.^13–15^ These static parameters do not capture the haemodynamic forces driving progressive aortic degeneration, which can result in delayed or missed TEVAR—particularly in patients with borderline morphology in whom diameter-based thresholds alone may not reliably predict subsequent enlargement.^16–18^

Four-dimensional flow magnetic resonance imaging (4D flow MRI) enables non-invasive assessment of aortic haemodynamics and is a promising adjunct to anatomical imaging.^19^^,^^20^ Previous studies have linked complex flow patterns or entry-related flow metrics with adverse aortic remodelling.^21–23^ However, clinical adoption has been limited by technically demanding workflows and the lack of standardized, interpretable parameters. Physiologically meaningful, simple, and readily applicable haemodynamic metrics are needed in routine clinical practice.

Mechanistically, we hypothesized that systolic FL-dominant flow acts as an upstream haemodynamic driver of early aortic progression by elevating intraluminal pressure and wall stress, complementing static anatomical assessment.^21^^,^^23^^,^^24^ As a step towards improving early risk stratification, we investigated whether systolic flow-based indices derived from 4D flow MRI can predict early aortic enlargement in patients with uTBAD.

## METHODS

### Study design and ethics

This single-centre observational cohort study, conducted at a tertiary referral centre for aortic disease, investigated haemodynamic predictors of early aortic enlargement in patients with uTBAD using 4D flow MRI. Institutional Review Board approval was obtained (Dokkyo Medical University; Protocol No. R-66-10J), and all procedures complied with the Declaration of Helsinki. Written informed consent was obtained from all participants.

Three complementary analyses were performed: (1) correlation between 4D flow MRI haemodynamic parameters and the first-year aortic growth rate; (2) group comparisons between aortic progression and stable patients; and (3) exploratory multivariable logistic regression adjusting for baseline MAD. The 12-month window captures the period when pre-emptive TEVAR decisions are most relevant and when TEVAR most effectively promotes aortic remodelling, with efficacy diminishing beyond 1 year.^25^ A sensitivity analysis reclassified patients with TEVAR into the progression group to reflect real-world clinical decision-making.

### Study setting and clinical care

Acute uTBAD was initially managed with OMT, including strict blood pressure and heart rate control, with conservative management after haemodynamic stabilization.^10^^,^^26^ TEVAR was considered through shared decision-making for high-risk features including rapid aortic enlargement (≥5 mm on short-interval CT) or MAD ≥40 mm at onset or follow-up within the first year; pre-emptive TEVAR was deferred when extensive supra-aortic debranching (≥2 vessels) was required. Beyond 1 year, intervention followed guideline-based thresholds. Throughout the study period, management was overseen by a consistent surgical team using the same protocols, with no major shifts in indication thresholds.

### Patient selection

Patients with acute uTBAD diagnosed by contrast-enhanced CT between August 2015 and April 2025 were screened (Stanford type B dissection without rupture or malperfusion).^26^ Inclusion criteria were patent or partially thrombosed FL, haemodynamic stabilization, and availability of baseline and follow-up CT images. Exclusion criteria were total FL thrombosis or ulcer-like projection, concomitant thoracic aortic aneurysm, fewer than 2 CT images, or contraindications to MRI.

The observation period was the interval from dissection onset to the last CT performed for growth rate analysis, censored at TEVAR or at 12 months, whichever came first. The median observation period until censoring was 0.87 (IQR 0.29-1.10) years.

### CT measurement and aortic growth rate

Contrast-enhanced CT followed institutional protocols, with surveillance scans during the initial hospitalization and at approximately 3, 6, and 12 months, with additional imaging as clinically indicated. Baseline assessments included primary entry location (aortic zone), dissection extent, and entry diameter. Coaxial reformatted images were used to measure MAD, true lumen (TL), and FL diameters. Established CT predictors of late enlargement (MAD at onset ≥40 mm, primary entry tear >10 mm, FL diameter >22 mm, FL at the lesser curvature, and partial FL thrombosis) were also assessed. Follow-up measurements were performed at corresponding anatomical levels. The aortic growth rate (mm/year) was calculated by ordinary least squares (OLS) regression of serial MAD measurements as a per-patient summary measure within the observation window (onset to censoring at TEVAR or 12 months); the median number of contributing CT time points was 4 (IQR 4-5). All measurements were performed by a single experienced observer.

### Definition of aortic growth progression

Patients were classified into a progression group or a stable group based on CT-measured changes in MAD within the first year.

**Figure 1.**
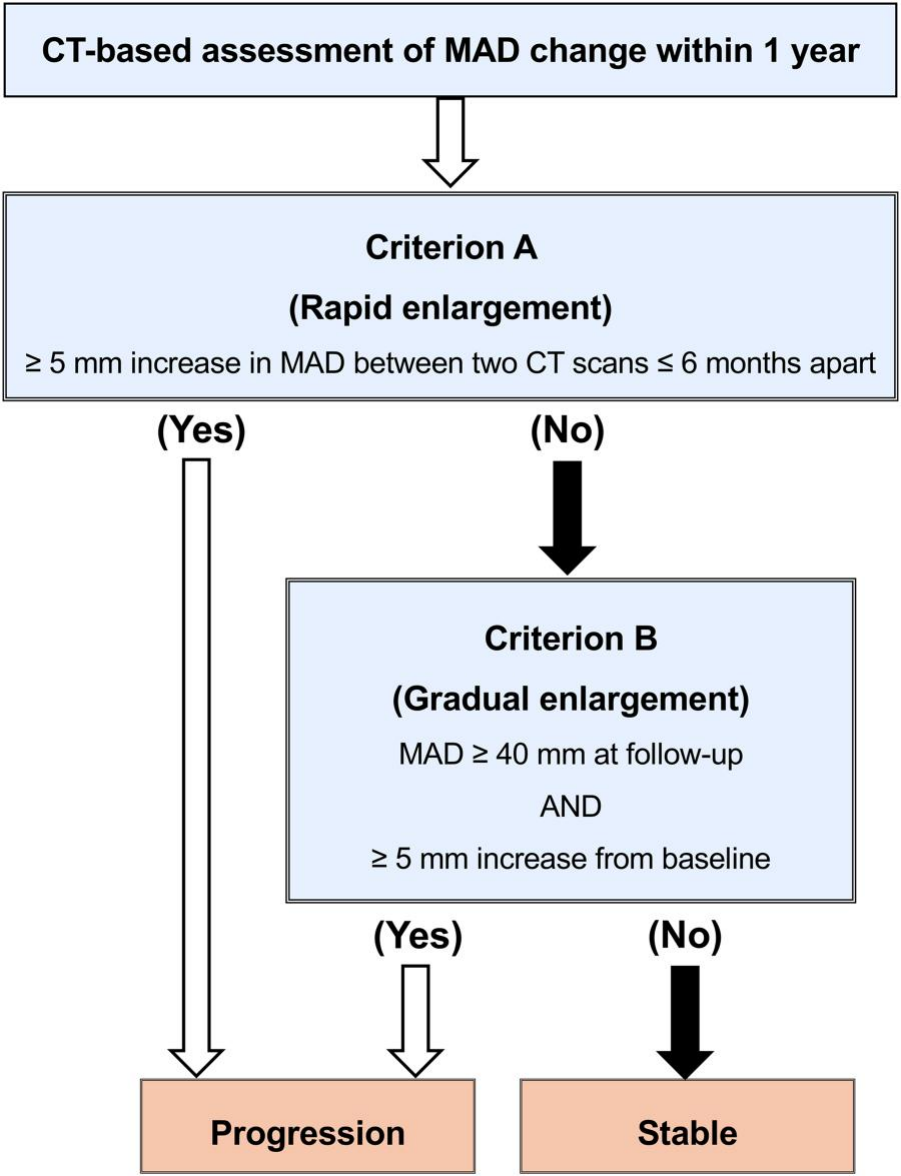
Definition of Aortic Progression. CT-based criteria for first-year aortic progression. Progression requires criterion A and/or B; patients meeting neither are stable. Abbreviations: CT, computed tomography; MAD, maximum aortic diameter

Aortic growth progression was defined as meeting either of the following criteria (**Figure 1**):

- Criterion A (rapid enlargement): ≥5-mm increase between 2 consecutive CT images obtained ≤6 months apart.
- Criterion B (gradual enlargement): MAD ≥ 40 mm at follow-up and ≥5-mm increase from baseline.

Patients who met neither criterion were considered stable.

### 4D flow MRI acquisition and flow parameter analysis

4D flow MRI was performed once during the early phase of dissection management, at a median of 42 days after onset (IQR 22-78), either during the initial hospitalization or at a scheduled outpatient visit following discharge, once haemodynamic stabilization had been achieved. MRI was acquired using a 3.0-T scanner with respiratory motion compensation and electrocardiographic gating, covering the thoracic aorta without contrast medium. Data were analysed using iTFlow (version 3.4; Cardio Flow Design Inc., Tokyo, Japan). The region of interest was selected according to the primary entry location, typically in the descending thoracic aorta (zones 4-5), or at the aortic valve level when the entry site was indeterminate. TL and FL flow rates (L/min) were quantified, with positive values representing antegrade and negative retrograde flow (**Figure S1**).

Four systolic haemodynamic parameters were derived: (1) FL flow index (0-1), (2) FL gross flow (L/min), (3) TL gross flow (L/min), (4) FL flow range (L/min).

Representative flow waveforms are shown in **Figure S2**. Formal mathematical definitions are provided in the Supplementary equation.

### Data collection and statistical analysis

Clinical and imaging data were retrospectively extracted from electronic records. Continuous variables are presented as median (IQR) and categorical variables as numbers (%). Spearman’s rank correlation was the primary method for assessing associations between haemodynamic parameters and the aortic growth rate, with Pearson’s correlation applied after exclusion of IQR-defined outliers. We obtained 95% CIs by bias-corrected and accelerated bootstrap resampling (10 000 iterations). Correlation strength was interpreted using conventional thresholds (0.1-0.3, weak; 0.3-0.5, moderate; and >0.5, strong). Group comparisons used the Mann-Whitney *U* test and the χ^2^/Fisher’s exact test, as appropriate. Sensitivity analyses reassigned patients with TEVAR to the progression group. An exploratory multivariable logistic regression tested the association between FL flow index and CT-based progression, adjusting for baseline MAD; with 16 events, the model was limited to these 2 standardized predictors (per 1-SD increase; events-per-variable ≈8). We additionally performed an exploratory Cox proportional hazards analysis of the time from onset to CT-based progression, censored at TEVAR or at 12 months, whichever occurred first.

Analyses were performed in Python (version 3.11); *P* < .05 was considered significant. All 36 patients had complete data for the primary variables; entry tear size was missing in 4 patients (unmeasurable on CT), affecting only its descriptive comparison. Given the exploratory design, no correction for multiple comparisons was applied, and no formal sample size calculation was performed.

## RESULTS

### Patient demographics

**Figure 2.**
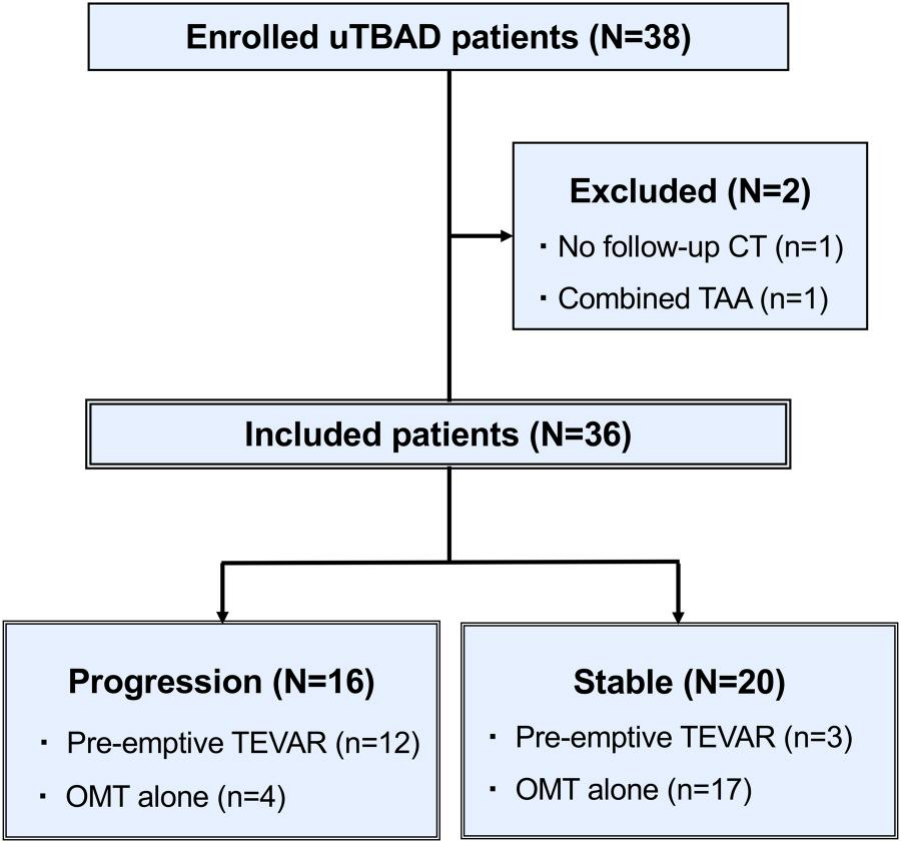
Study Flow Diagram. Abbreviations: uTBAD, uncomplicated type B aortic dissection; CT, computed tomography; TAA, thoracic aortic aneurysm; OMT, optimal medical therapy; TEVAR, thoracic endovascular aortic repair

Of 38 identified patients with uTBAD, 36 were included (**Figure 2**); 2 were excluded, one with a concomitant descending thoracic aortic aneurysm, and one scheduled for pre-emptive TEVAR at presentation without follow-up CT. Baseline characteristics are summarized in **Table 1**. The cohort was 83% male with a median age of 55.5 years, and one patient had Marfan syndrome. Most primary entry tears occurred in zone 3; 4 were invisible on CT, and 4D flow MRI identified the entry in 2.

**Table 1.** Baseline Clinical Characteristics Stratified by CT-Based Aortic Progression

Variable	Total	Progression	Stable
	(*n* = 36)	(*n* = 16)	(*n* = 20)
Age (years)	55.5 [47.8-63.5]	52.0 [46.8-58.2]	60.0 [51.2-71.2]
Male sex, *n* (%)	30 (83.3)	15 (93.8)	15 (75.0)
Comorbidity, *n* (%)			
Hypertension	26 (72.2)	11 (68.8)	15 (75.0)
Chronic kidney disease	6 (16.7)	2 (12.5)	4 (20.0)
Marfan syndrome	1 (2.8)	0	1 (5.0)
Cerebrovascular disease	0	0	0
Atrial fibrillation	0	0	0
Primary entry location, *n* (%)			
Zone 3	21 (58.3)	11 (68.8)	10 (50.0)
Zone 4	9 (25.0)	4 (25.0)	5 (25.0)
Zone 5	4 (11.1)	1 (6.2)	3 (15.0)
Unidentified	2 (5.6)	0	2 (10.0)

Values are median [IQR] or *n* (%). Progression follows CT-based aortic growth criteria within the first year after onset.

Abbreviation: CT, computed tomography.

### Aortic progression and clinical management

During the first year, 20 patients remained stable, and 16 met the CT-based progression criteria (2 criterion A, 10 criterion B, and 4 both; **Table S1**); 16 of the stable patients had observation periods >6 months. Overall, 15 patients underwent TEVAR within the first year, with no deaths. Of the 16 progression patients, 4 (all criterion B) did not undergo pre-emptive TEVAR: 3 owing to patient preference and one (zone 5 entry tear) owing to concerns regarding spinal cord ischaemia. Conversely, 3 stable-group patients underwent pre-emptive TEVAR for high-risk features, including an initial aortic diameter ≥40 mm, Marfan syndrome with a borderline diameter of 39 mm, or significant TL compression without malperfusion.

### Correlation between 4D flow MRI flow parameters and aortic growth rate

The OLS-derived aortic growth rate ranged from −5.5 to 199.9 mm/year, with 3 patients showing negative growth (FL regression). All individual diameter-time regression plots are provided in **Supplementary material M1.** 

**Figure 3.**
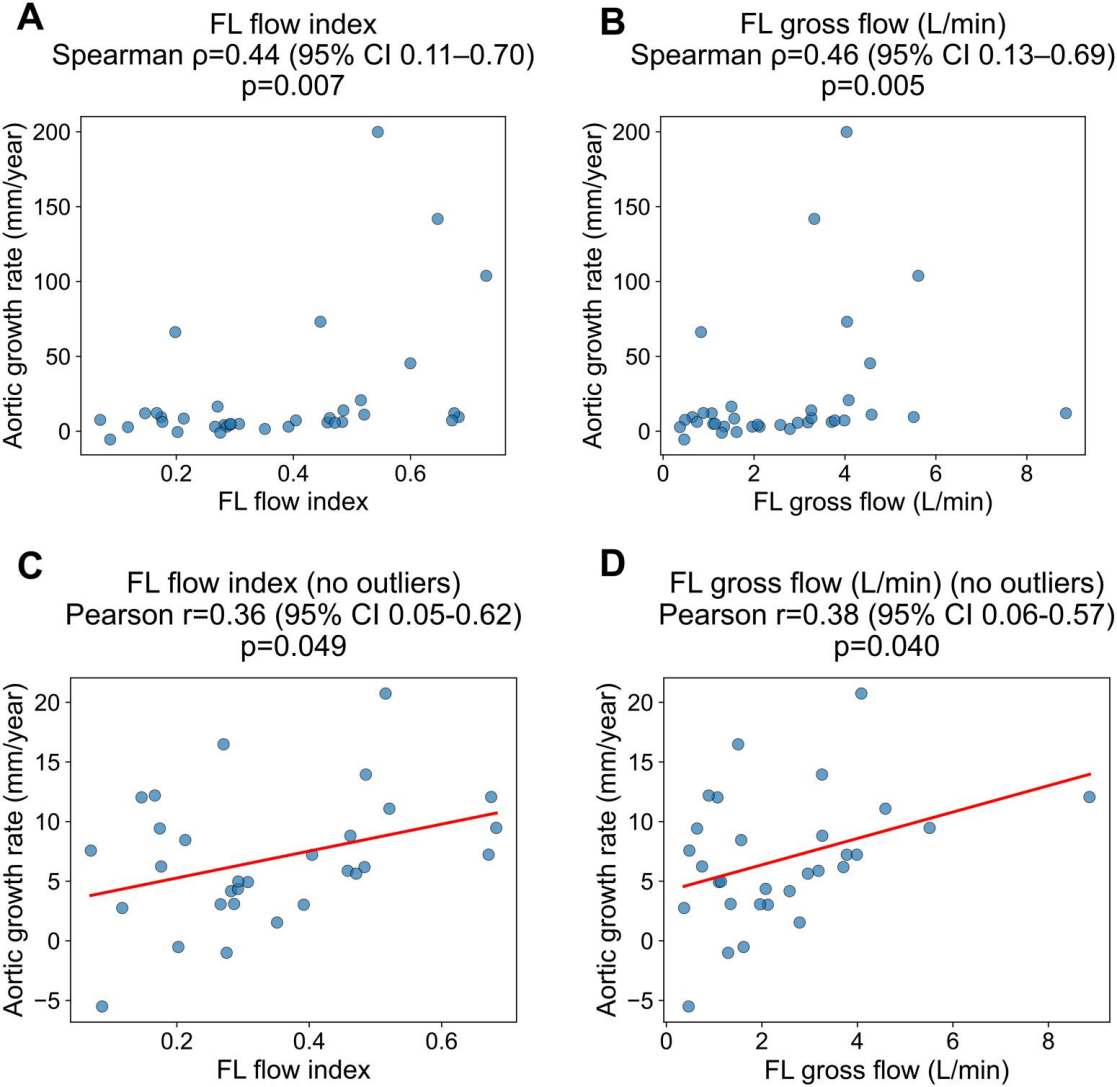
Correlation Between the 4D Flow MRI-Derived Flow Parameters and Early Aortic Growth Rate. (A, B) Aortic growth rate vs systolic FL flow index (A) and FL gross flow (B), Spearman correlation with bootstrap 95% CI (*n* = 36). (C, D) Corresponding Pearson correlations after outlier exclusion with bootstrap 95% CI (*n* = 30). Abbreviations: 4D flow MRI, four-dimensional flow magnetic resonance imaging; FL, false lumen

Among all haemodynamic indices, the FL flow index and FL gross flow showed moderate positive correlations with the aortic growth rate using Spearman’s rank correlation (**Figure 3A and B**). These findings remained robust on Pearson’s correlation analysis after excluding statistical outliers (*n* = 30): FL flow index and FL gross flow (**Figure 3C and D**). Conversely, the TL gross flow and FL flow range showed no significant correlations (**Figure S3**).

### Comparison of anatomical and haemodynamic parameters between groups

**Table 2** summarizes CT-derived anatomical features and 4D flow MRI-derived haemodynamic parameters between groups. Baseline diameter and CT-based risk features did not differ. At follow-up, the progression group exhibited a larger MAD and higher growth rate and, on MRI, demonstrated a higher FL flow index and FL gross flow.

**Table 2.** Anatomical and Haemodynamic Differences Between Patients With and Without CT-Based Aortic Progression

Variable	Total	Progression	Stable	*P* value
	(*n* = 36)	(*n* = 16)	(*n* = 20)	
CT measurement				
MAD onset (mm)	33.3 [31.5-37.8]	37.1 [31.6-38.5]	32.0 [31.4-35.1]	.13
MAD latest follow-up (mm)	39.7 [36.8-43.4]	43.5 [40.3-47.4]	37.4 [34.9-38.4]	<.01
Aortic growth rate (mm/y)	7.4 [4.3-12.6]	13.1 [9.3-52.3]	4.6 [3.0-6.2]	<.01
Entry size (mm)	8.7 [6.4-11.2]	8.7 [6.4-11.2]	8.4 [6.6-11.2]	.99
MAD onset ≥40 mm, *n* (%)	6 (16.7)	4 (25.0)	2 (10.0)	.64
Entry size >10 mm, *n*/*N* (%)	12/32 (37.5)	6/16 (37.5)	6/16 (37.5)	1.00
Partial thrombosis, *n* (%)	15 (41.7)	6 (37.5)	9 (45.0)	.65
FL at lesser curvature, *n* (%)	4 (11.1)	1 (6.2)	3 (15.0)	.61
FL >22 mm at onset, *n* (%)	6 (16.7)	3 (18.8)	3 (15.0)	1.00
4D flow MRI parameter				
FL flow index	0.33 [0.21-0.49]	0.50 [0.26-0.61]	0.29 [0.20-0.39]	.04
FL gross flow (L/min)	2.35 [1.13-3.83]	3.66 [1.55-4.20]	1.79 [1.09-2.83]	.03
TL gross flow (L/min)	5.18 [4.25-6.87]	5.03 [3.81-8.07]	5.18 [4.76-6.85]	.53
FL flow range (L/min)	4.66 [3.00-6.30]	6.07 [3.49-6.89]	4.30 [2.69-5.62]	.15

Values are median [IQR] or *n* (%). Entry size and entry size >10 mm are unavailable in 4 patients because the entry tear could not be identified on CT.

Abbreviations: 4D flow MRI, four-dimensional flow magnetic resonance imaging; CT, computed tomography; FL, false lumen; MAD, maximum aortic diameter; TL, true lumen.

In the exploratory multivariable logistic regression, the FL flow index remained associated with CT-based aortic progression after adjustment for baseline MAD, whereas baseline MAD did not reach statistical significance (**Table 3**).

**Table 3.** Exploratory Multivariable Logistic Regression for CT-Based Aortic Progression

Variable	OR	95% CI	*P* value
FL flow index (per 1-SD increase)	2.31	1.04-5.13	.039
Baseline MAD (per 1-SD increase)	1.73	0.81-3.69	.157

Exploratory logistic regression with CT-based progression as outcome, with predictors standardized (per 1-SD increase). Events-per-variable ≈ 8; McFadden pseudo-*R*^2^ = 0.155.

Abbreviations: CT, computed tomography; FL, false lumen; MAD, maximum aortic diameter; OR, odds ratio.

**Figure 4.**
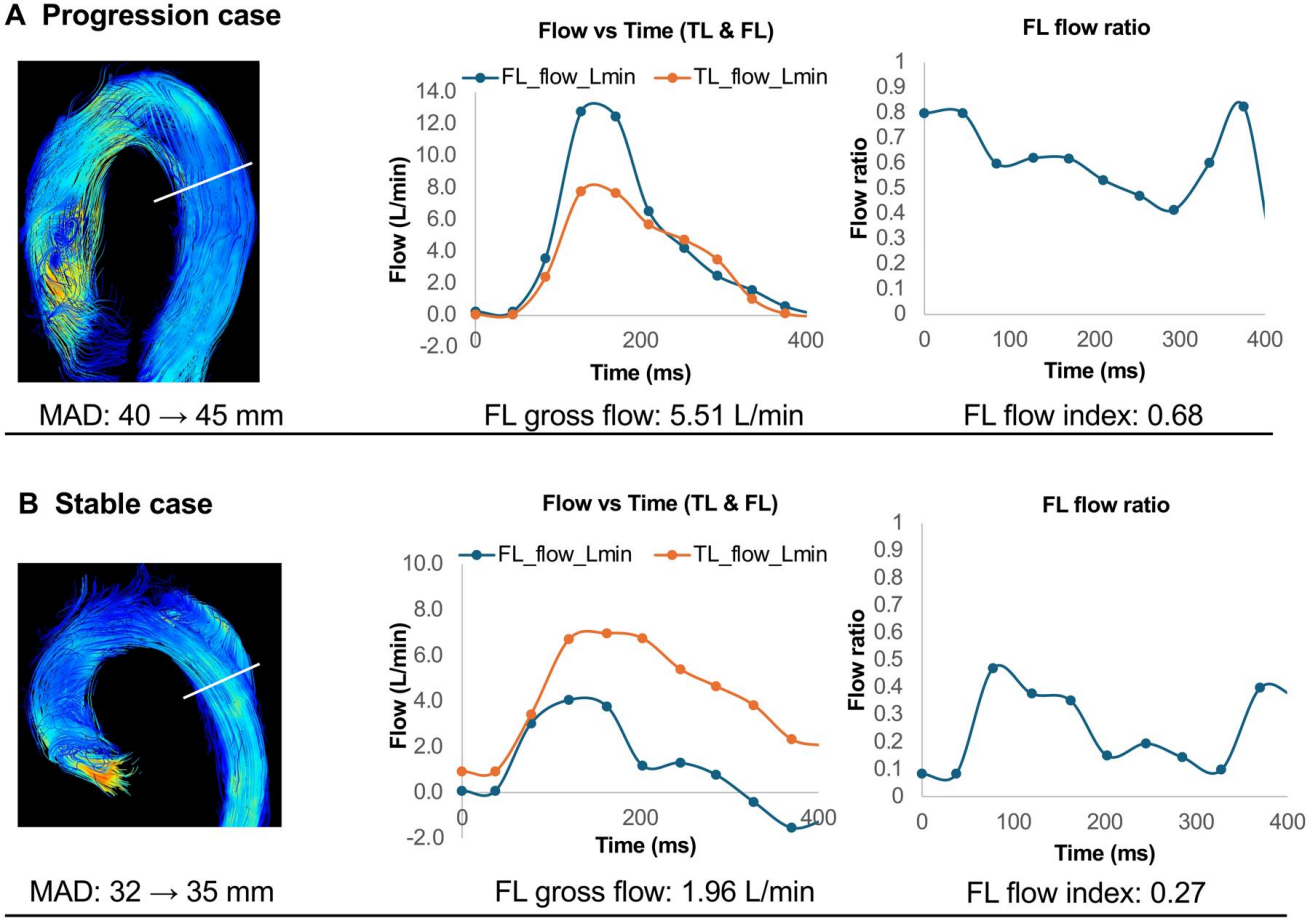
Representative 4D Flow MRI Haemodynamic Profiles in Patients With and Without CT-Based Aortic Progression. (A) Representative progression case. Streamline visualisation of aortic flow shows systolic FL-dominant flow at the quantification plane (white line), with higher FL flow index and FL gross flow and subsequent enlargement on follow-up CT. (B) Representative stable case. Predominantly TL flow with lower FL flow indices and no enlargement. Abbreviations: 4D flow MRI, four-dimensional flow magnetic resonance imaging; CT, computed tomography; FL, false lumen; MAD, maximum aortic diameter; TL, true lumen

A representative comparison of systolic flow waveforms is shown in **Figure 4**, demonstrating a greater systolic FL-dominant flow in patients with progression; flow waveforms for all cases are provided in **Supplementary material M2**. Group differences persisted in a sensitivity analysis reassigning TEVAR patients to the progression group (*n* = 19 vs 17; **Table S2**). On multivariable sensitivity analysis, FL flow index remained associated with progression (OR, 3.31; 95% CI, 1.16-9.45; *P* = 0.025), as did baseline MAD (OR, 4.51; 95% CI, 1.28-15.9; *P* = 0.019; **Table S3**). An exploratory Cox proportional hazards analysis produced concordant findings, with the FL flow index associated with progression after adjustment for baseline MAD (**Table S4**, **Figure S4**).

## DISCUSSION

This study investigated whether simple systolic haemodynamic indices derived from 4D flow MRI could predict early aortic enlargement in uTBAD. The principal finding was that the systolic FL-dominant flow, quantified by the FL flow index and FL gross flow, was associated with early aortic enlargement. On exploratory multivariable analysis, the FL flow index remained associated with aortic progression after adjustment for baseline MAD. This association remained consistent in sensitivity analyses incorporating patients who underwent TEVAR. Conversely, established CT-based anatomical predictors were similar between the groups, indicating that functional haemodynamic assessment provides prognostic information beyond static morphology.

These findings align with established fluid-dynamic principles. Greater systolic inflow into the FL increases pulsatile energy transfer, intraluminal pressure, and wall stress, limiting TL re-expansion and promoting FL dilatation.^24^ This haemodynamic burden may drive adverse remodelling before conventional diameter thresholds are reached.

The moderate correlation coefficients should be interpreted in the context of the nonlinear nature of aortic growth.^27^ Although OLS regression provides a simplified linear approximation, the correlation of single time-point systolic flow metrics with longitudinal enlargement underscores the biological relevance of these haemodynamic markers. These associations remained consistent after outlier exclusion, supporting their robustness.

Existing haemodynamic approaches each have practical limitations. Entry-based analyses, such as the FL ejection fraction,^21^ are susceptible to turbulence-related variability near the entry tear. Qualitative assessments of helical and vortical flow patterns provide valuable physiological insights but lack standardized quantification.^22^ Diastolic flow metrics are technically challenging because of low velocities and gating sensitivity.^23^ Building on the concept that increased FL inflow drives adverse remodelling,^24^ the present approach focuses on simplified systolic indices. Because the 4D flow MRI acquisition window typically covers approximately 80% of the cardiac cycle and diastolic flow is minimal in uTBAD, systolic haemodynamics likely represent the principal driver of early FL pressurization. Restricting analysis to systole therefore improves reproducibility and clinical applicability.

An important consideration is whether the FL flow index predicts future progression or reflects remodelling already in progress. Because MRI was performed at a median of 42 days after onset, some remodelling may have preceded imaging. In patients with overt enlargement at MRI, a high FL flow index primarily confirms an ongoing adverse trajectory often clinically evident. Conversely, in patients not yet meeting CT-based progression criteria, a high FL flow index may identify those at risk of subsequent enlargement in whom closer surveillance with shorter CT follow-up intervals is warranted. Supporting this latter role, the FL flow index remained associated with progression after adjustment for baseline MAD, and 9 of 16 patients with progression had an MAD below 40 mm at the time of MRI. These indices should therefore be interpreted as tools for early risk stratification rather than direct indicators for intervention.

Although MRI was performed at a single time point, serial 4D flow MRI represents a promising surveillance avenue. Initially hostile flow patterns may stabilize over time, whereas favourable patterns could deteriorate if new entry tears develop or partial thrombosis resolves. Prospective studies are needed to validate thresholds and evaluate longitudinal haemodynamic monitoring.

### Limitations

The single-centre retrospective design with a modest sample size may limit generalizability. 4D flow MRI was performed at a single time point and at varying intervals after dissection onset, reflecting routine clinical practice in which uniform early-phase imaging is not always feasible and precluding assessment of temporal haemodynamic changes. Technical challenges in acquisition and post-processing, with potential variability across MRI vendors and software, may also affect reproducibility. CT measurements were performed by a single observer; therefore, interobserver variability was not assessed. Blood pressure—an important contributor to aortic enlargement—was managed by guideline-directed OMT but was not systematically recorded for analysis.

Statistically, the aortic growth rate was estimated by OLS regression; nonlinear or mixed-effects modelling was not feasible at this sample size and should be pursued in larger studies. Given the small number of events, the time-to-event analysis was exploratory, and our findings demonstrate associations rather than absolute risk estimates. The exploratory multivariable model was restricted to baseline MAD as the sole covariate.

## CONCLUSION

These hypothesis-generating findings indicate that systolic FL-dominant flow quantified by the FL flow index and FL gross flow was associated with early aortic enlargement in patients with uTBAD. These simple, reproducible indices reflect key drivers of adverse remodelling not captured by conventional CT morphology alone and may complement CT imaging in identifying patients at elevated risk of early progression. Prospective, multicentre studies are warranted to validate these findings and clarify the role of haemodynamic biomarkers in uTBAD management.

## Supplementary Material

ivag198_Supplementary_Data

## Data Availability

The data underlying this article will be shared on reasonable request to the corresponding author.

## ABBREVIATIONS

CT: 4D flow
MRI: four-dimensional flow magnetic resonance imaging
CT: computed tomography
FL: false lumen
MAD: maximum aortic diameter
OLS: ordinary least squares
OMT: optimal medical therapy
OR: odds ratio
TEVAR: thoracic endovascular aortic repair
TL: true lumen
uTBAD: uncomplicated type B aortic dissection
