# Supplementary material for: Haemodynamic Predictors of Early Aortic Growth in Uncomplicated Type B Dissection: A Cohort Study
Source: Interdiscip Cardiovasc Thorac Surg. 2026 Jul 8;41(7):ivag198. doi: 10.1093/icvts/ivag198 (PMC13408290; doi:10.1093/icvts/ivag198)

# Supplementary Material M1

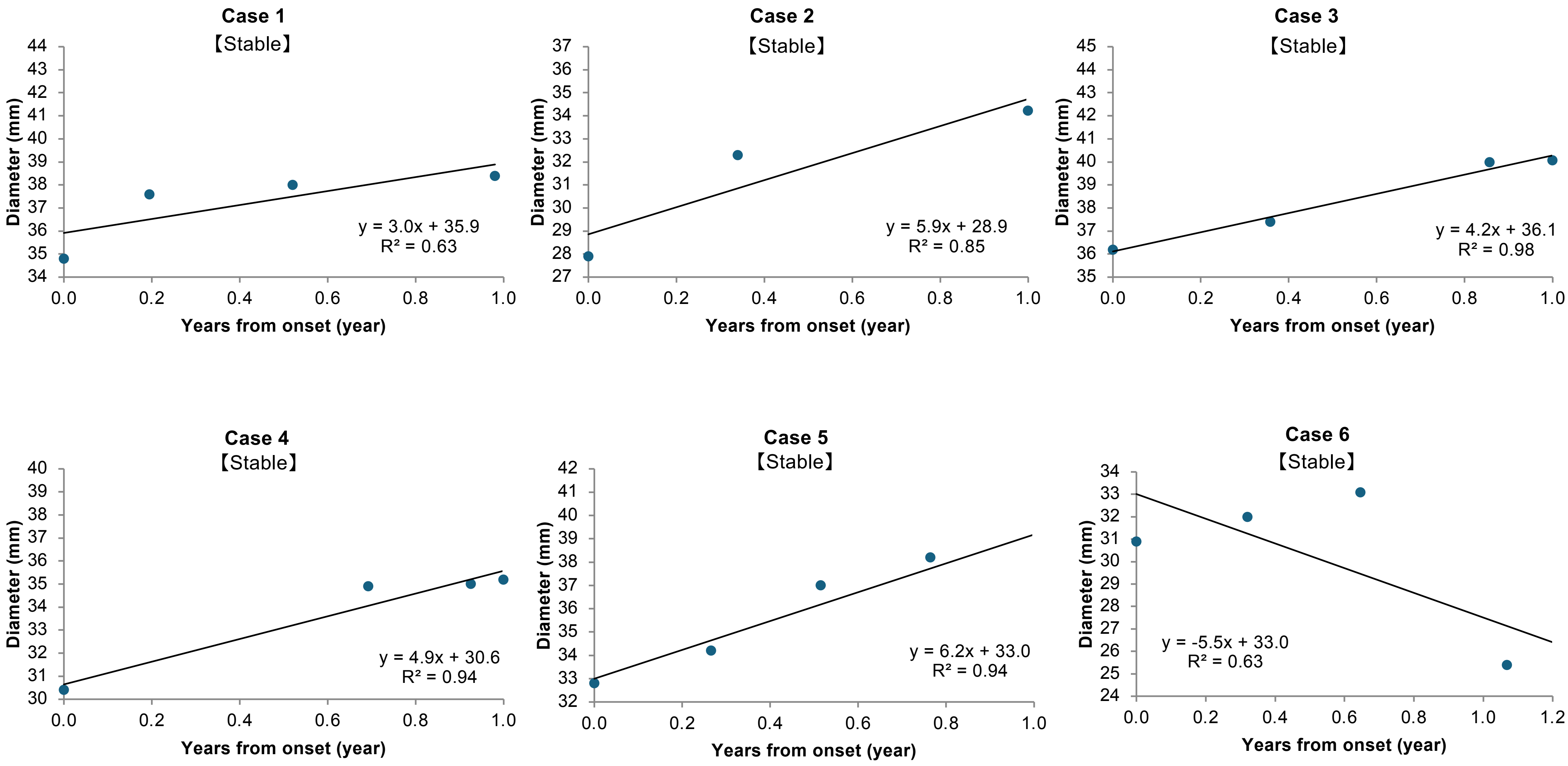
Case 1
【Stable】
y = 3.0x + 35.9
R² = 0.63
Case 2
【Stable】
y = 5.9x + 28.9
R² = 0.85
Case 3
【Stable】
y = 4.2x + 36.1
R² = 0.98
Case 4
【Stable】
y = 4.9x + 30.6
R² = 0.94
Case 5
【Stable】
y = 6.2x + 33.0
R² = 0.94
Case 6
【Stable】
y = -5.5x + 33.0
R² = 0.63
Diameter (mm)
Years from onset (year)

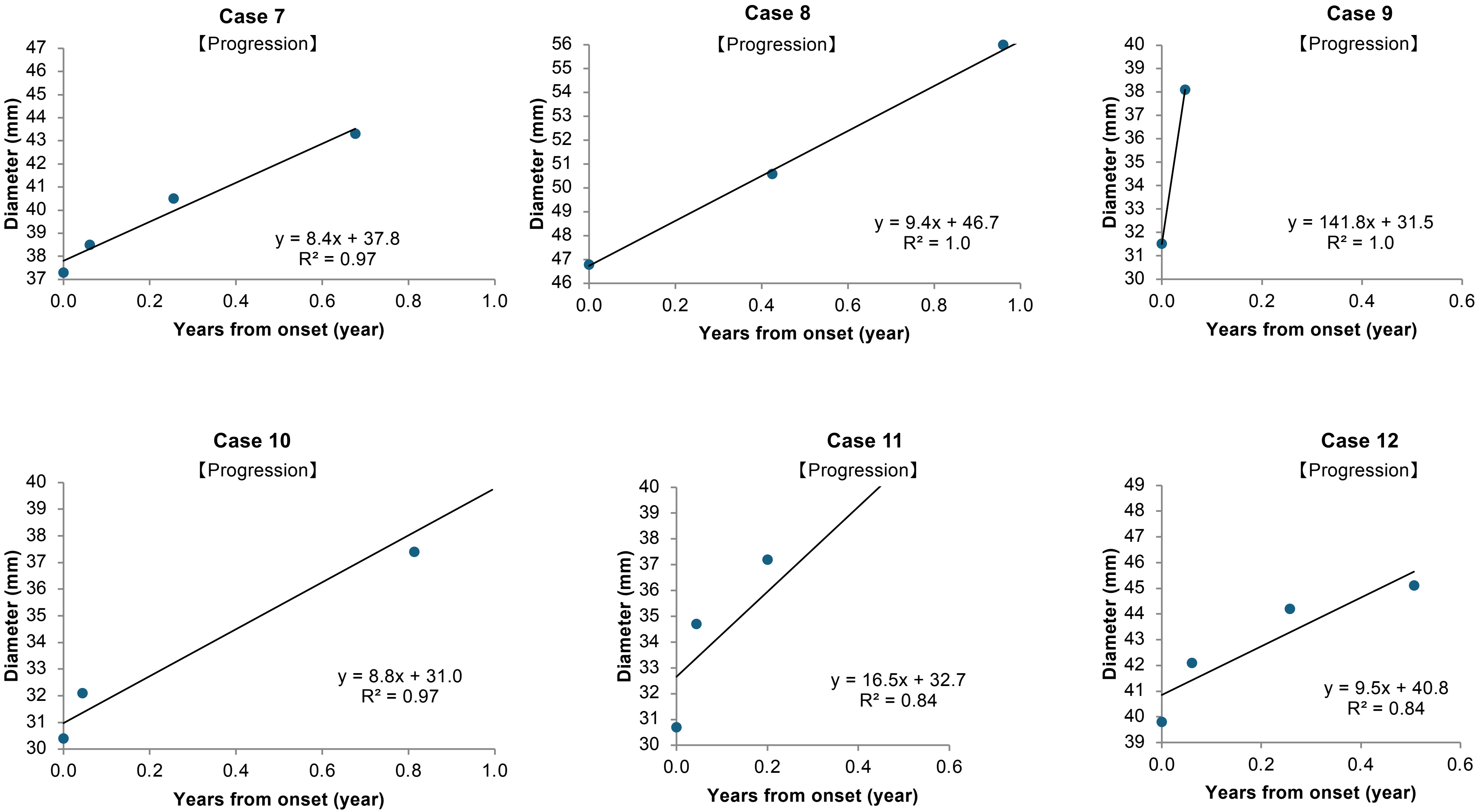
Case 7
【Progression】
y = 8.4x + 37.8
R² = 0.97
Case 8
【Progression】
y = 9.4x + 46.7
R² = 1.0
Case 9
【Progression】
y = 141.8x + 31.5
R² = 1.0
Case 10
【Progression】
y = 8.8x + 31.0
R² = 0.97
Case 11
【Progression】
y = 16.5x + 32.7
R² = 0.84
Case 12
【Progression】
y = 9.5x + 40.8
R² = 0.84
Diameter (mm)
Years from onset (year)

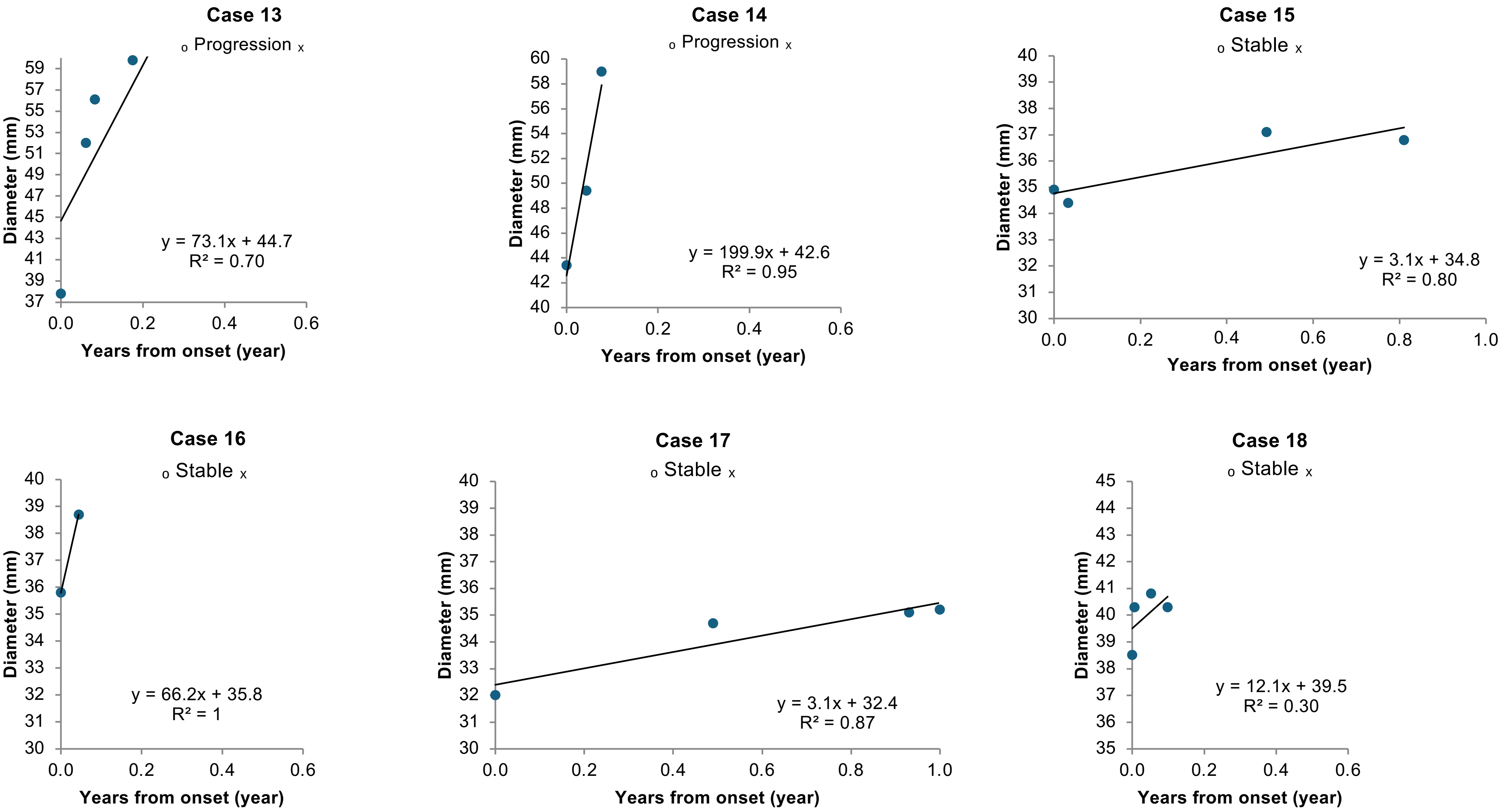
Case 13
o Progression x
Diameter (mm)
Years from onset (year)
y = 73.1x + 44.7
R² = 0.70
Case 14
o Progression x
Diameter (mm)
Years from onset (year)
y = 199.9x + 42.6
R² = 0.95
Case 15
o Stable x
Diameter (mm)
Years from onset (year)
y = 3.1x + 34.8
R² = 0.80
Case 16
o Stable x
Diameter (mm)
Years from onset (year)
y = 66.2x + 35.8
R² = 1
Case 17
o Stable x
Diameter (mm)
Years from onset (year)
y = 3.1x + 32.4
R² = 0.87
Case 18
o Stable x
Diameter (mm)
Years from onset (year)
y = 12.1x + 39.5
R² = 0.30

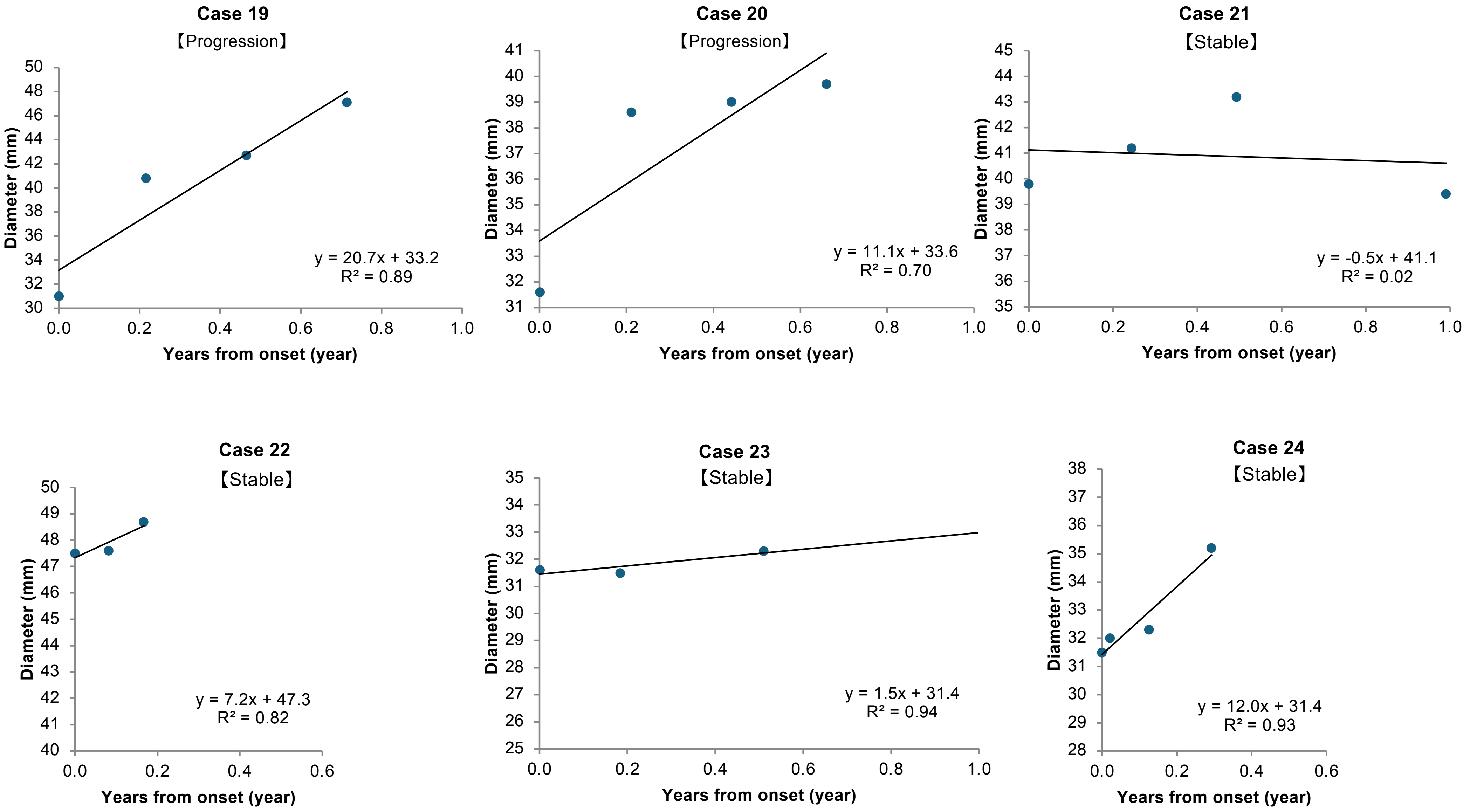

Case 19
【Progression】
y = 20.7x + 33.2
R² = 0.89
Diameter (mm)
Years from onset (year)
Case 20
【Progression】
y = 11.1x + 33.6
R² = 0.70
Diameter (mm)
Years from onset (year)
Case 21
【Stable】
y = -0.5x + 41.1
R² = 0.02
Diameter (mm)
Years from onset (year)
Case 22
【Stable】
y = 7.2x + 47.3
R² = 0.82
Diameter (mm)
Years from onset (year)
Case 23
【Stable】
y = 1.5x + 31.4
R² = 0.94
Diameter (mm)
Years from onset (year)
Case 24
【Stable】
y = 12.0x + 31.4
R² = 0.93
Diameter (mm)
Years from onset (year)

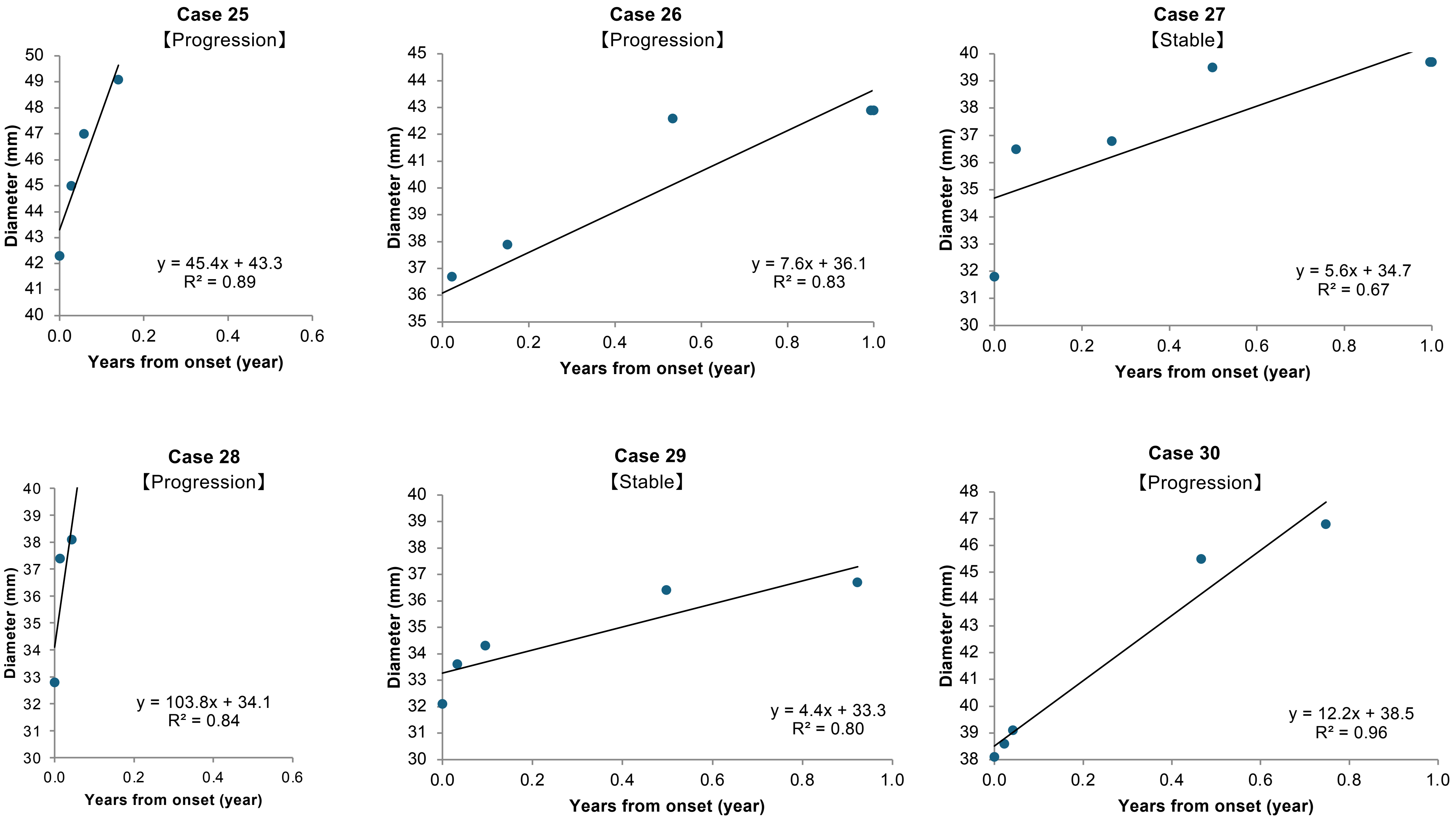
Case 25
【Progression】
y = 45.4x + 43.3
R² = 0.89
Diameter (mm)
Years from onset (year)
Case 26
【Progression】
y = 7.6x + 36.1
R² = 0.83
Diameter (mm)
Years from onset (year)
Case 27
【Stable】
y = 5.6x + 34.7
R² = 0.67
Diameter (mm)
Years from onset (year)
Case 28
【Progression】
y = 103.8x + 34.1
R² = 0.84
Diameter (mm)
Years from onset (year)
Case 29
【Stable】
y = 4.4x + 33.3
R² = 0.80
Diameter (mm)
Years from onset (year)
Case 30
【Progression】
y = 12.2x + 38.5
R² = 0.96
Diameter (mm)
Years from onset (year)

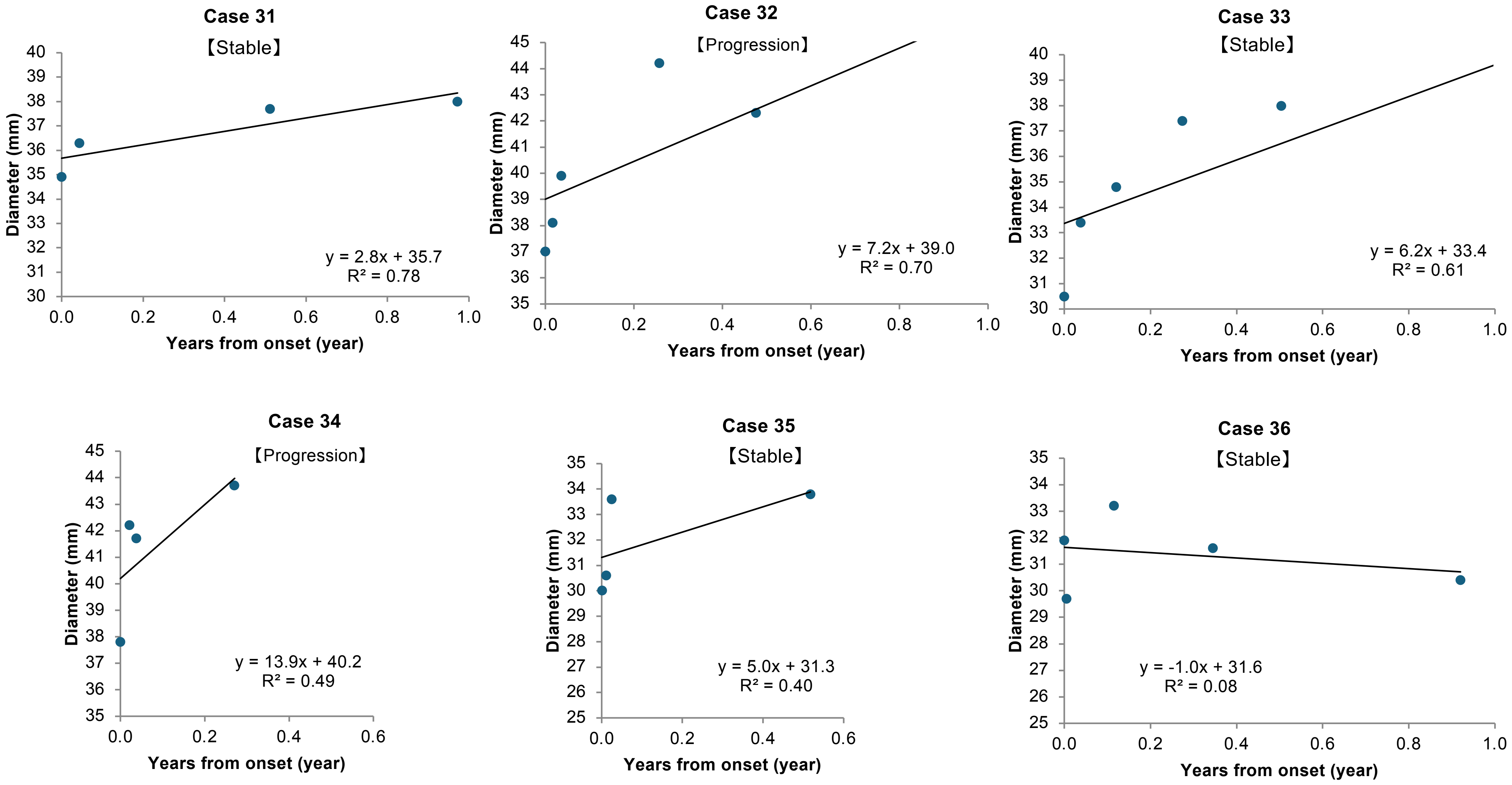

Case 31
【Stable】
y = 2.8x + 35.7
R² = 0.78
Diameter (mm)
Years from onset (year)
Case 32
【Progression】
y = 7.2x + 39.0
R² = 0.70
Diameter (mm)
Years from onset (year)
Case 33
【Stable】
y = 6.2x + 33.4
R² = 0.61
Diameter (mm)
Years from onset (year)
Case 34
【Progression】
y = 13.9x + 40.2
R² = 0.49
Diameter (mm)
Years from onset (year)
Case 35
【Stable】
y = 5.0x + 31.3
R² = 0.40
Diameter (mm)
Years from onset (year)
Case 36
【Stable】
y = -1.0x + 31.6
R² = 0.08
Diameter (mm)
Years from onset (year)

# Supplementary Material M2

**Case 1** (End-systolic time = 358 ms) [Stable]

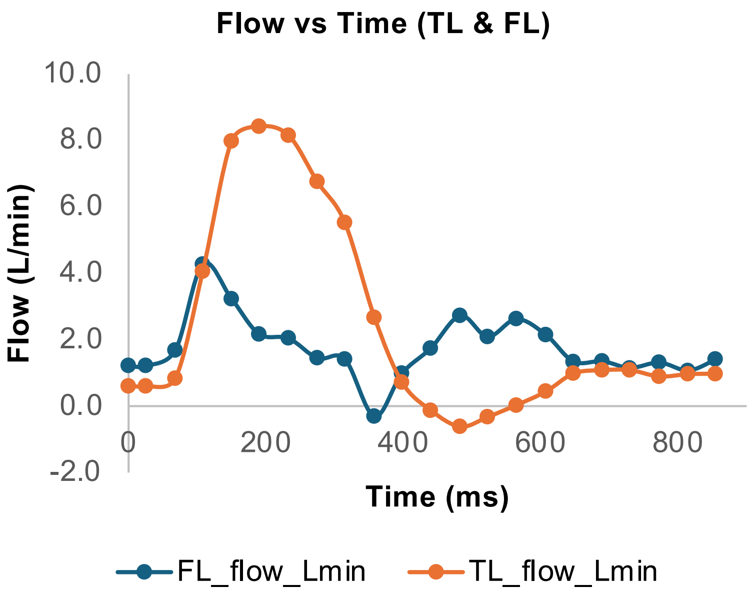


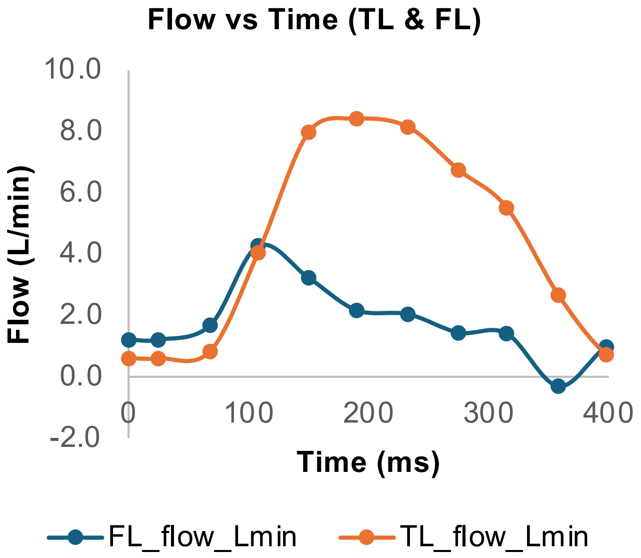


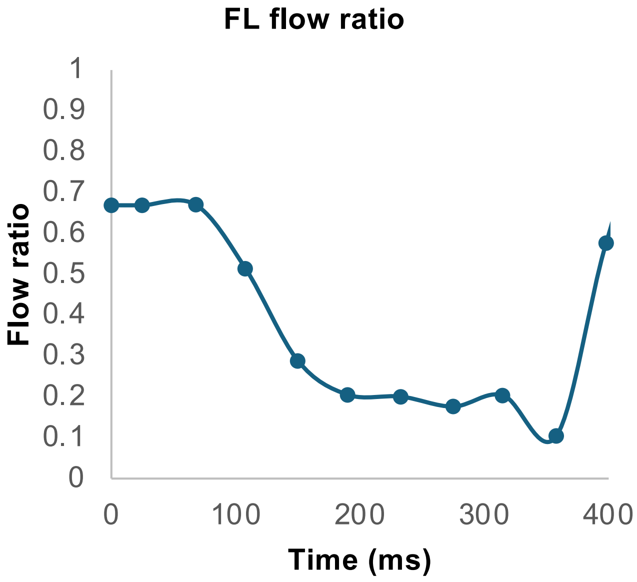


**Case 2** (End-systolic time = 365 ms) [Stable]

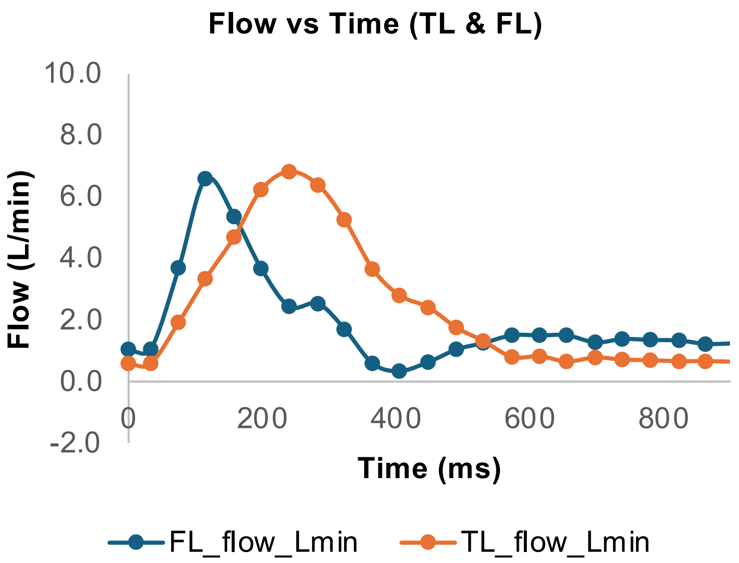


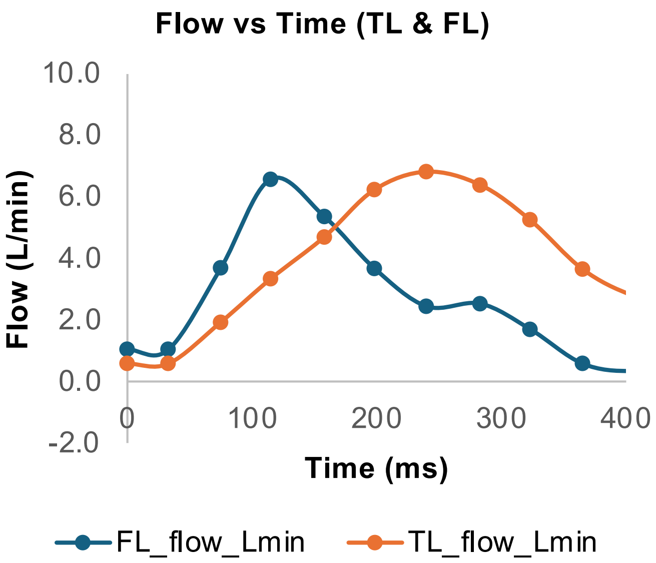


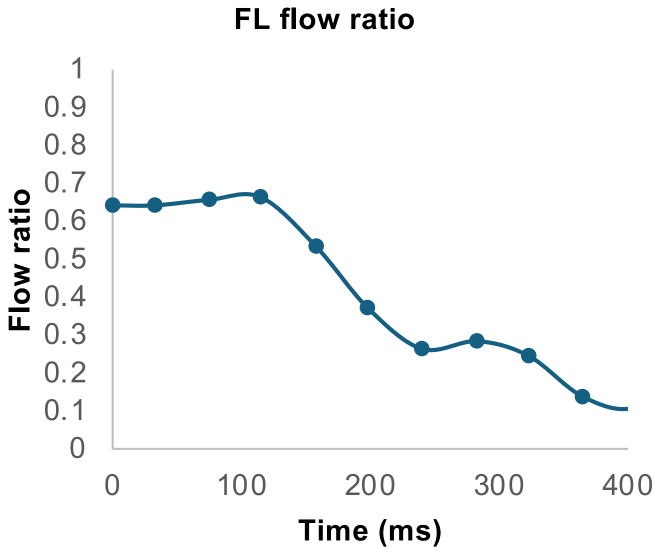

**Case 3** (End-systolic time = 383 ms) [Stable]

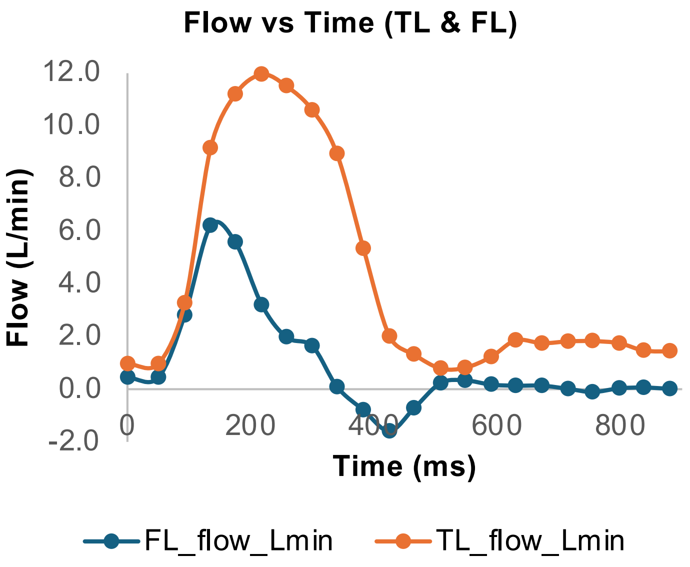


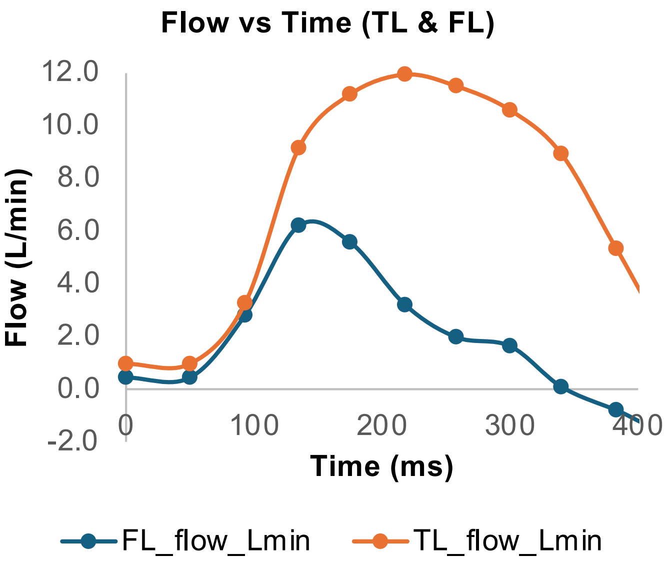


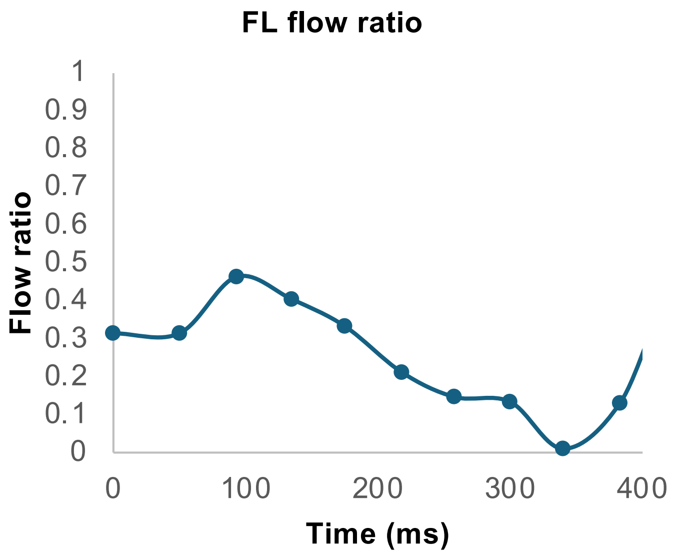


**Case 4** (End-systolic time = 348 ms) [Stable]

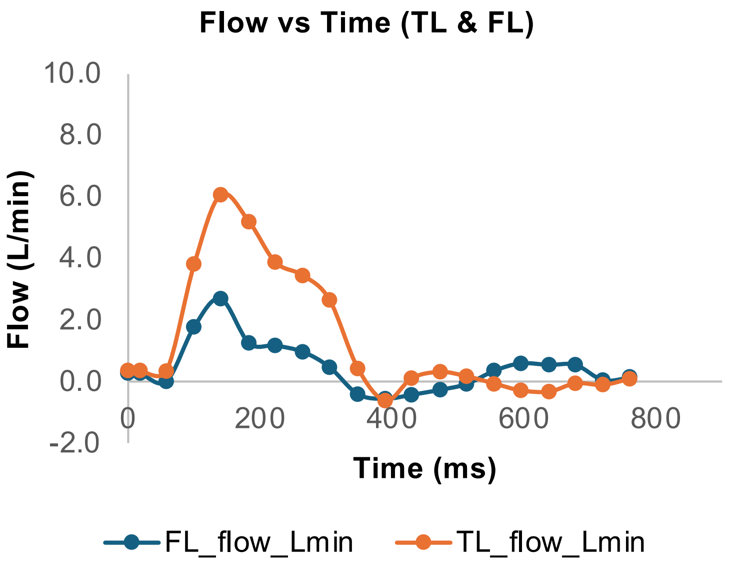


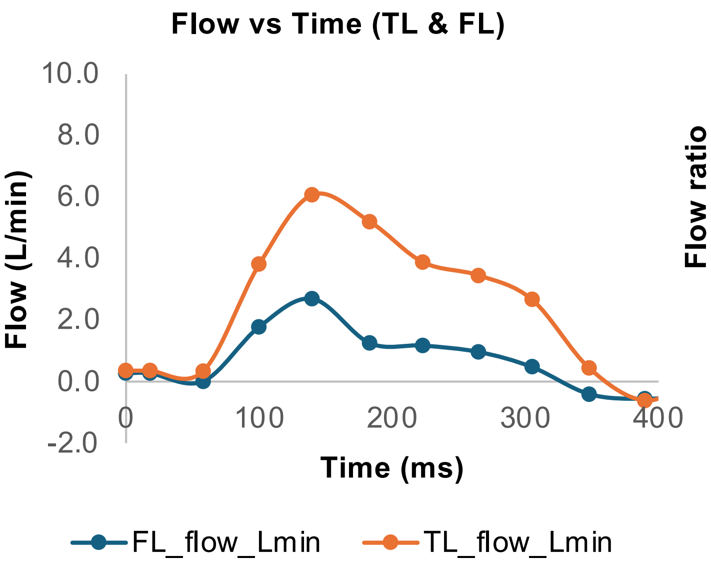


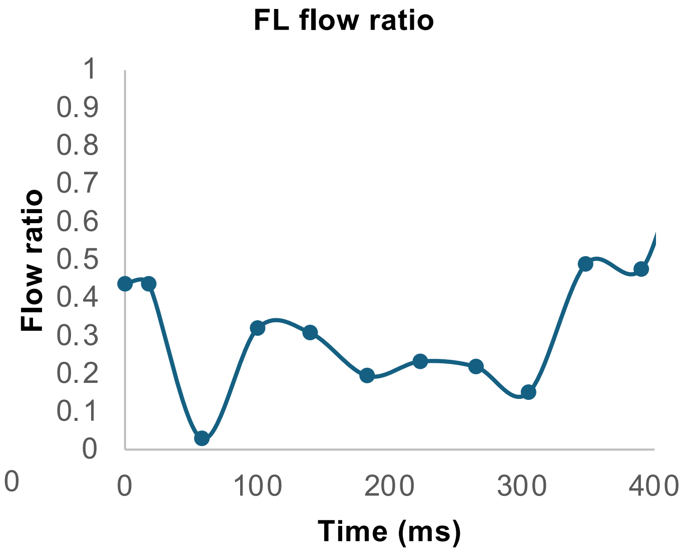

**Case 5** (End-systolic time = 360 ms) [Stable]

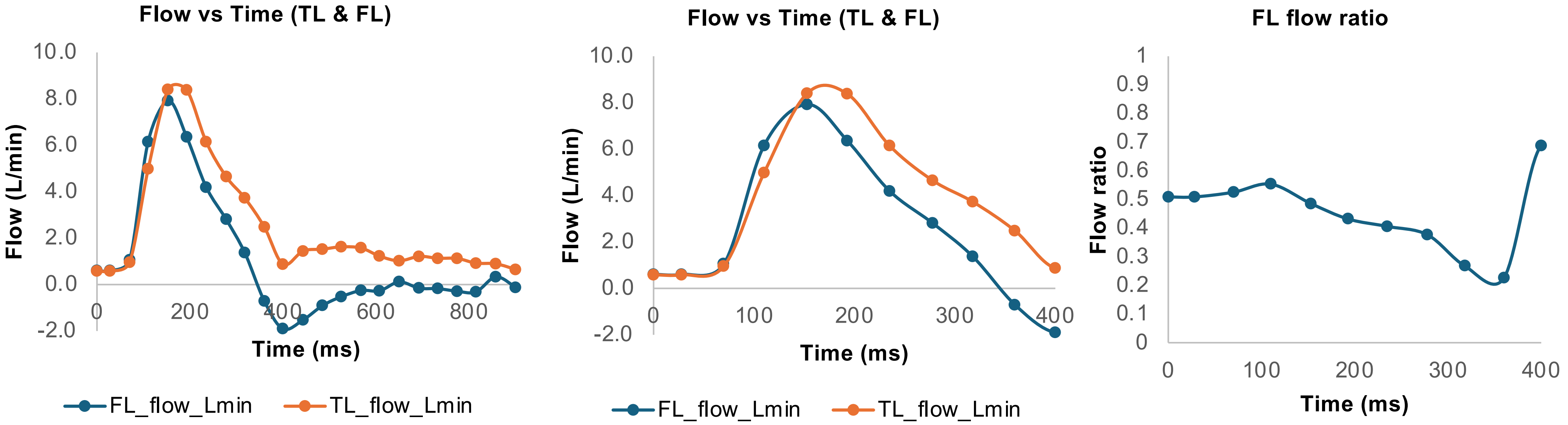


**Case 6** (End-systolic time = 348 ms) [Stable]

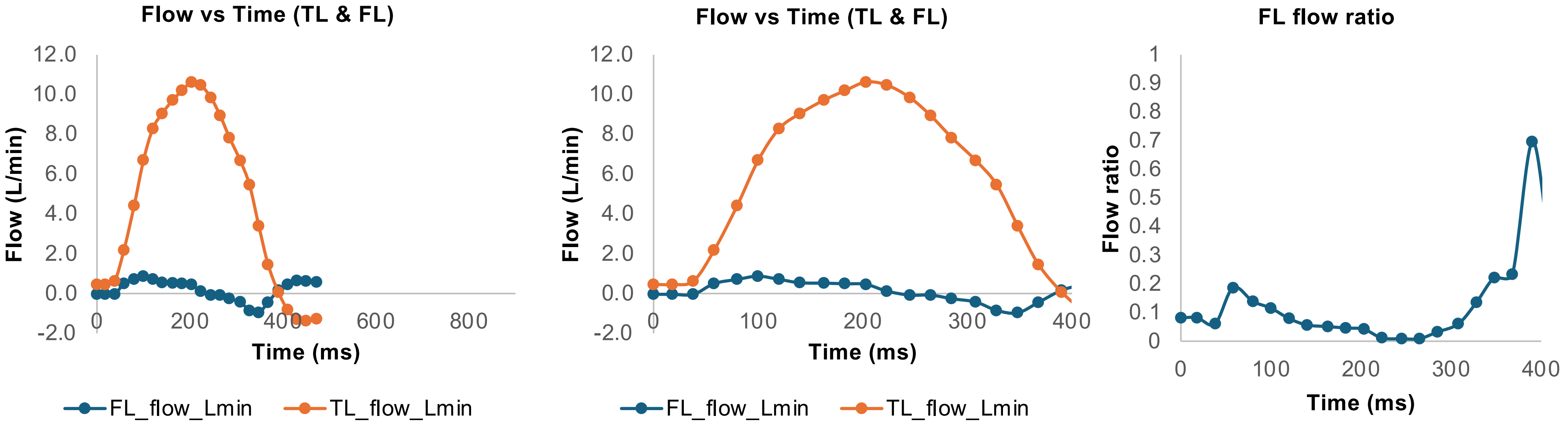

**Case 7** (End-systolic time = 345 ms) [Progression]

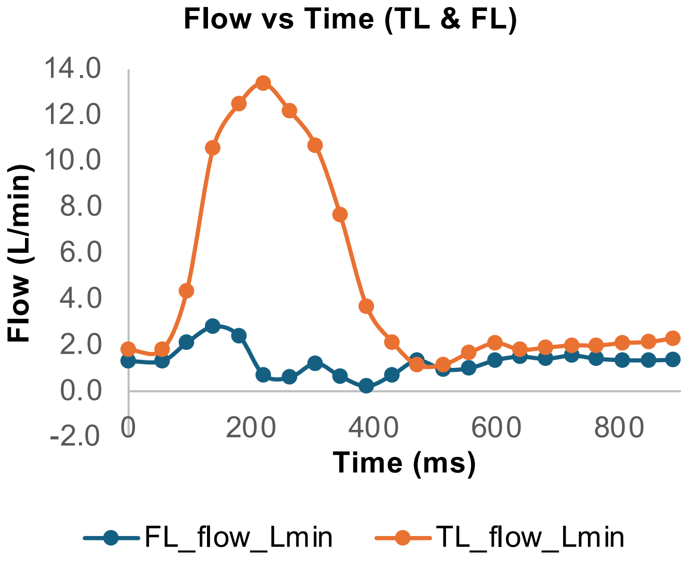


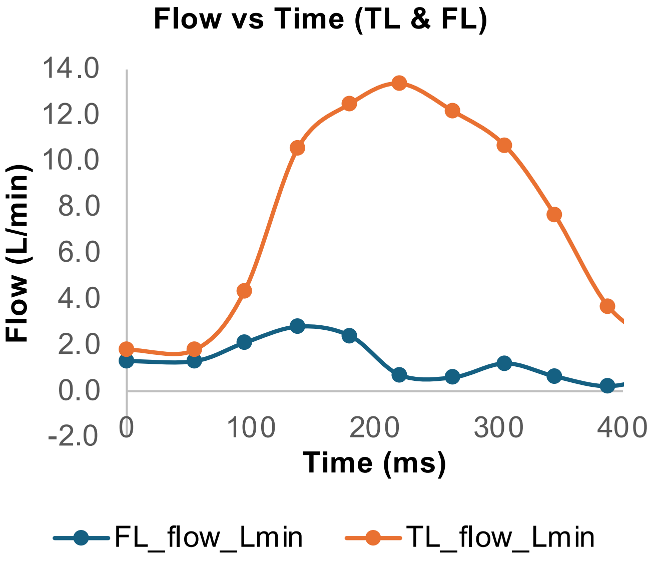


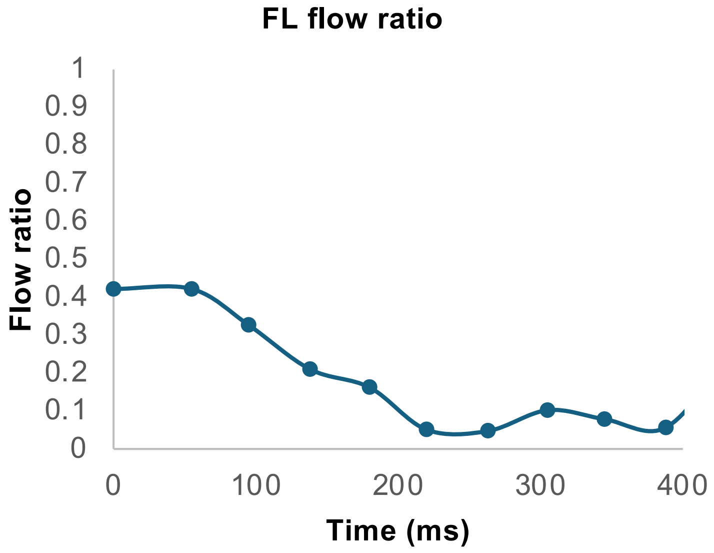


**Case 8** (End-systolic time = 383 ms) [Progression]

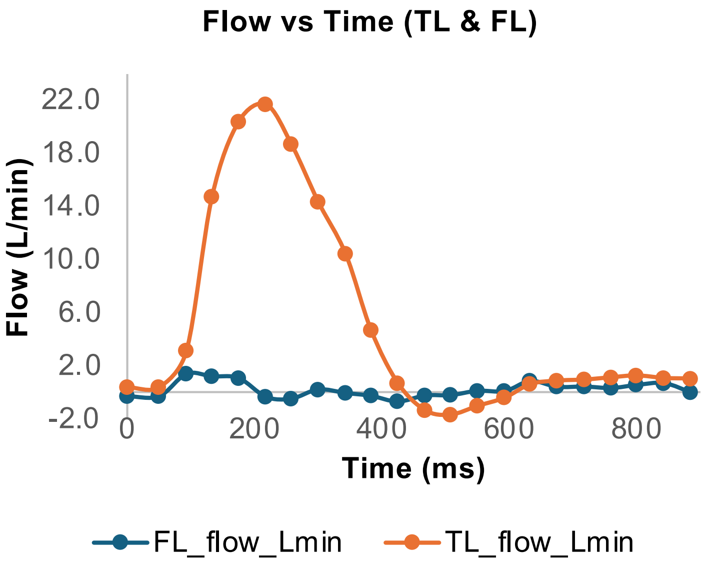


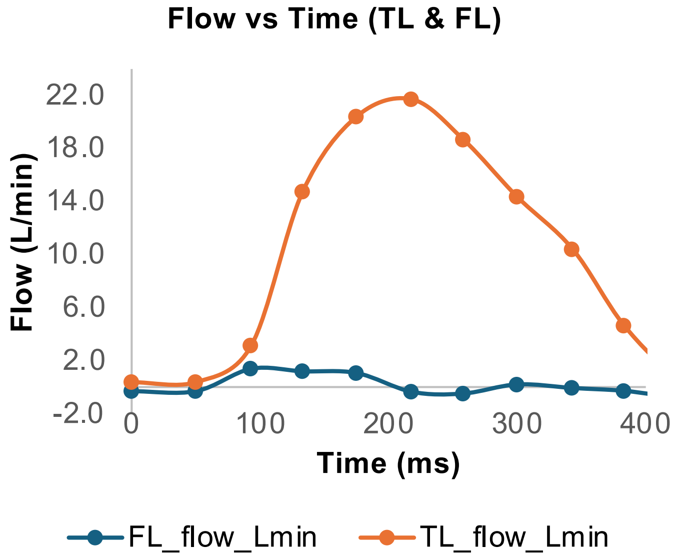


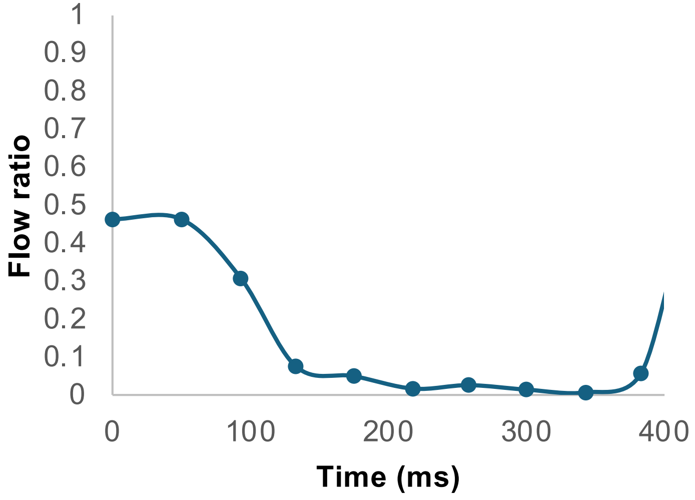

**Case 9** (End-systolic time = 363 ms) [Progression] + TEVAR

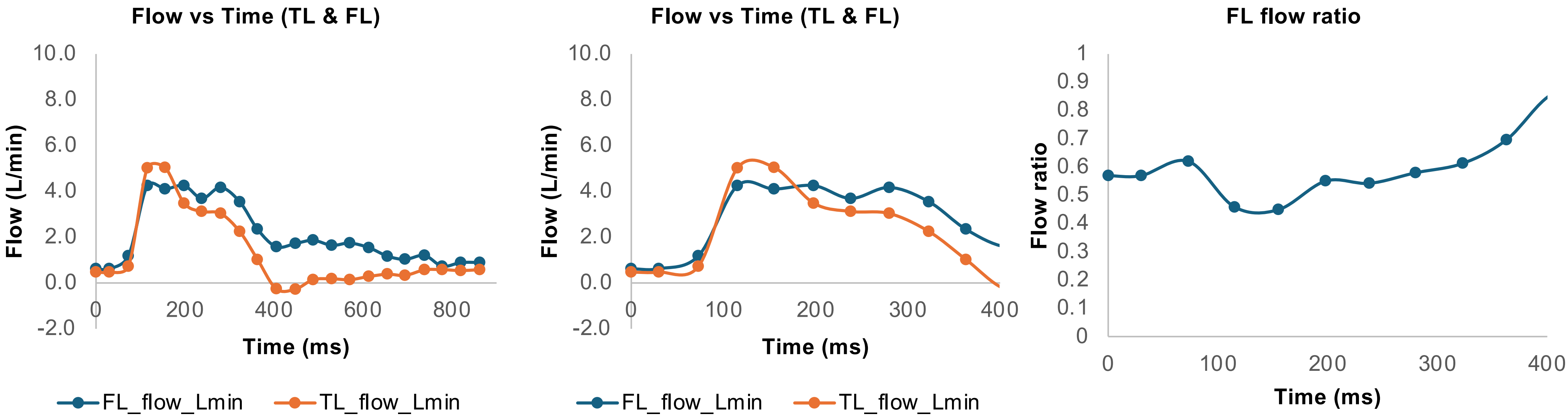


**Case 10** (End-systolic time = 305 ms) [Progression] + TEVAR

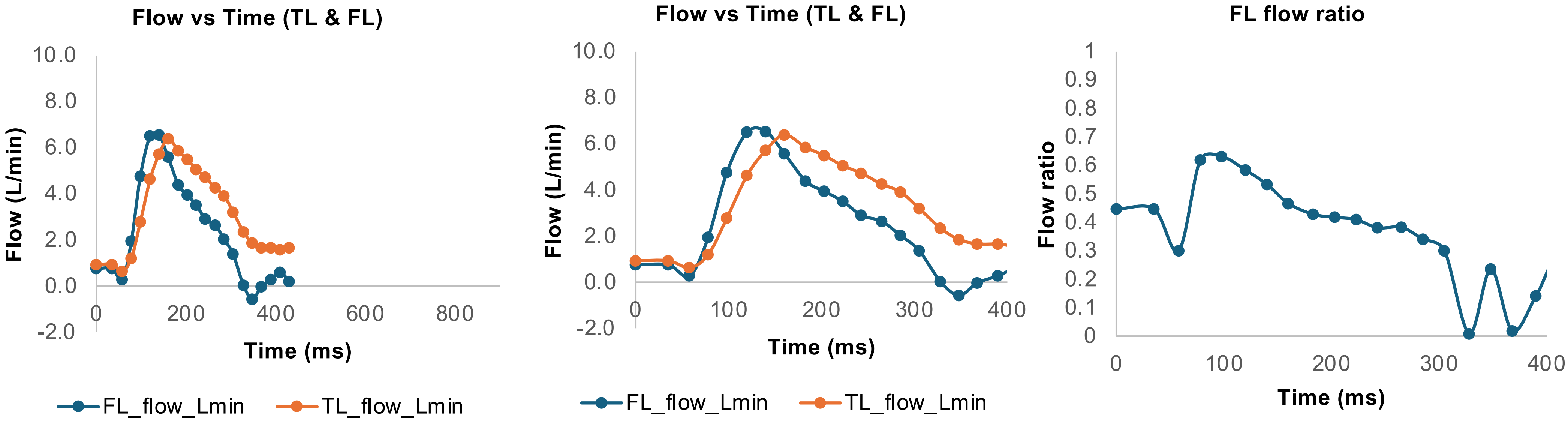

**Case 11** (End-systolic time = 388 ms) [Progression] + TEVAR

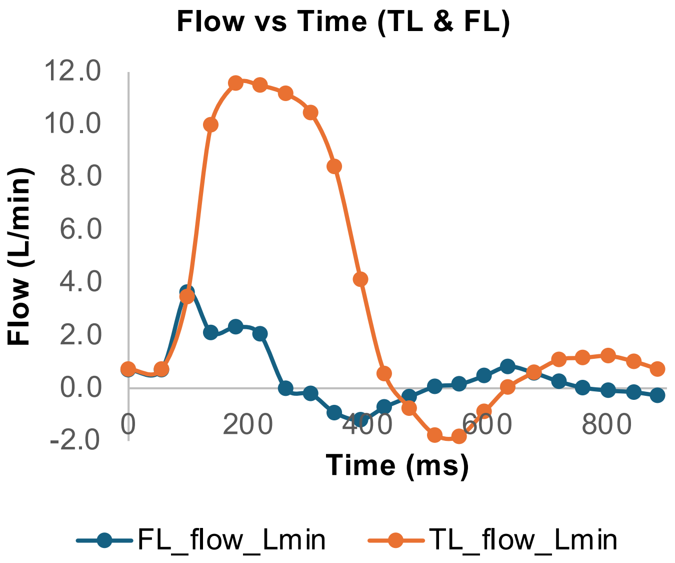


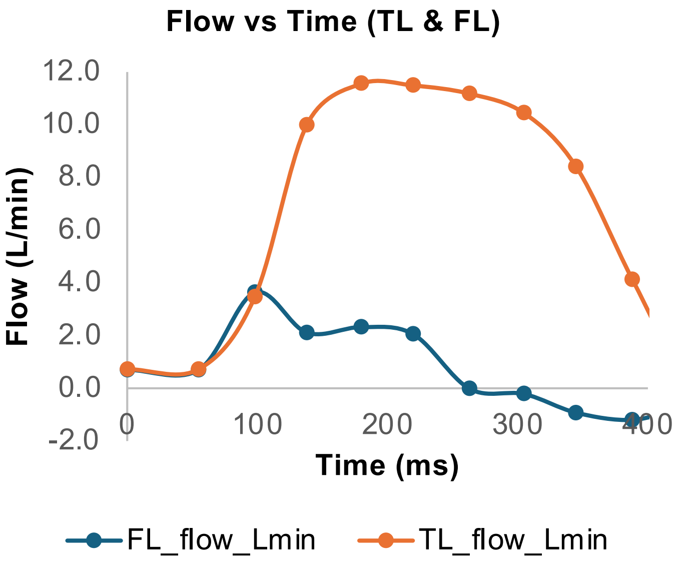


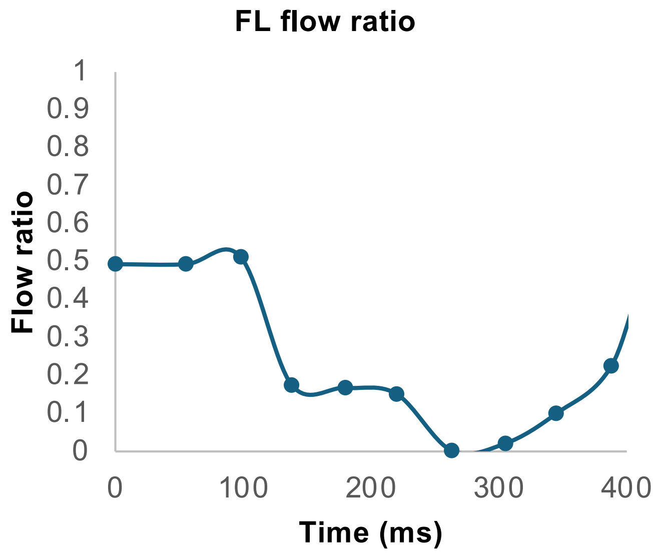


**Case 12** (End-systolic time = 335 ms) [Progression] + TEVAR

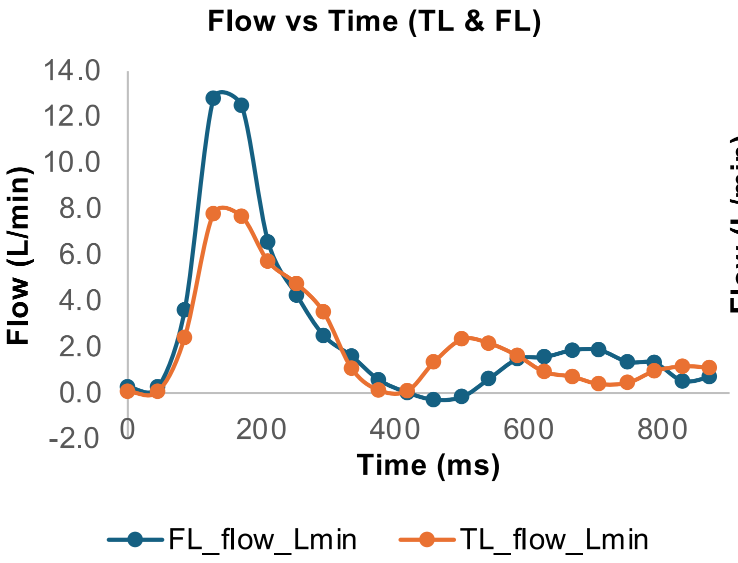


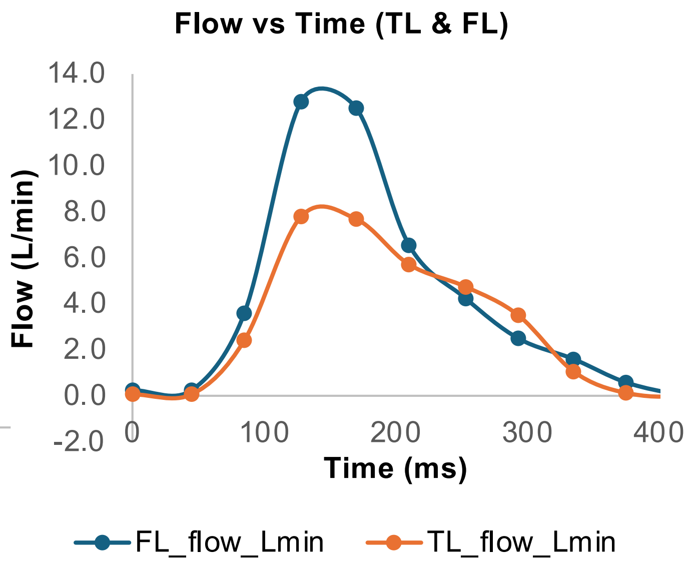


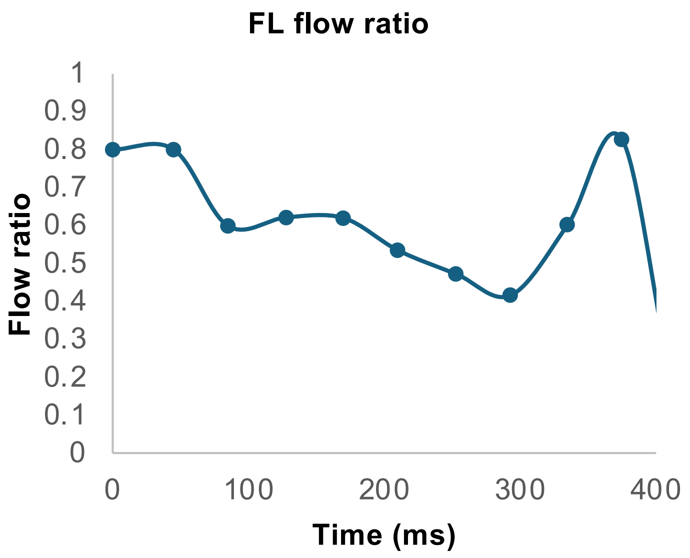

**Case 13** (End-systolic time = 383 ms) [Progression] + TEVAR

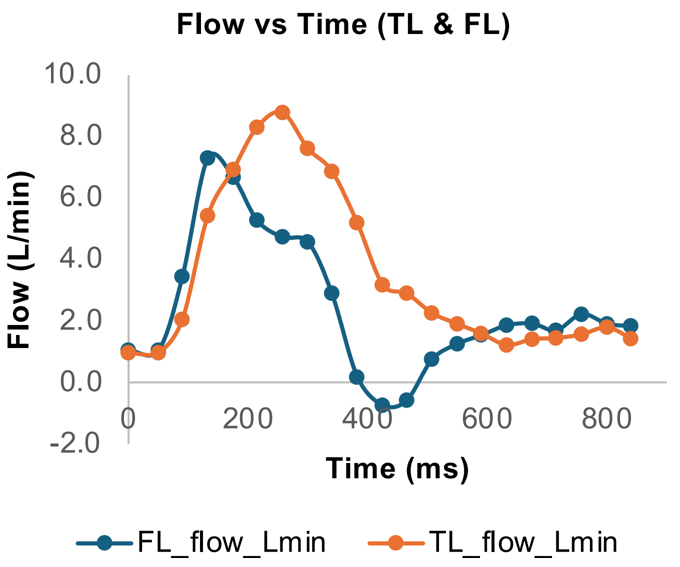


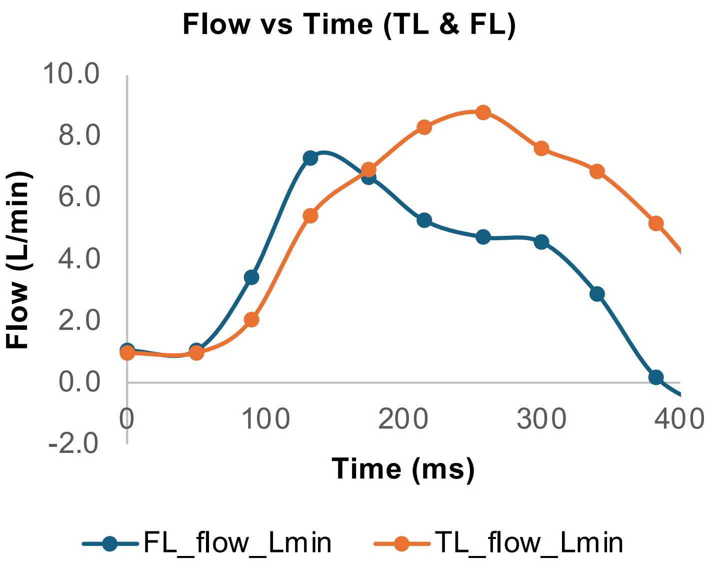


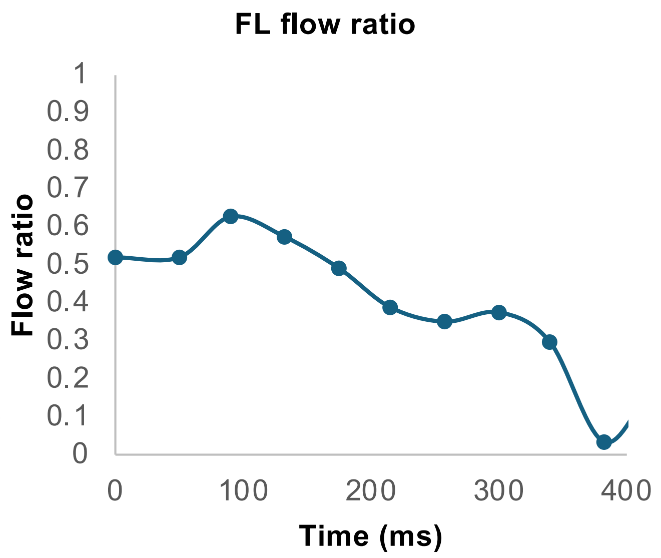


**Case 14** (End-systolic time = 318 ms) [Progression] + TEVAR

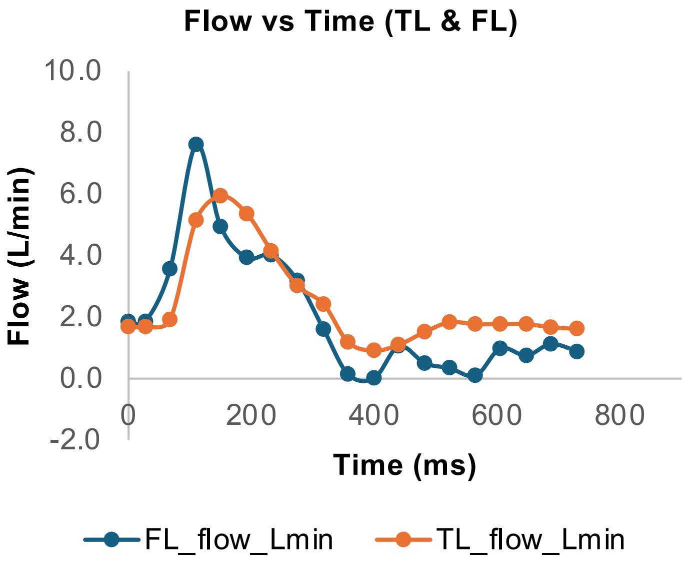


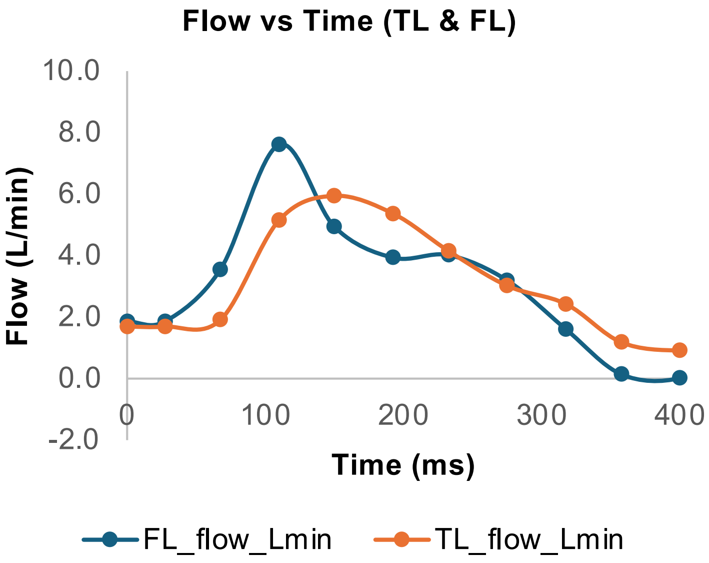


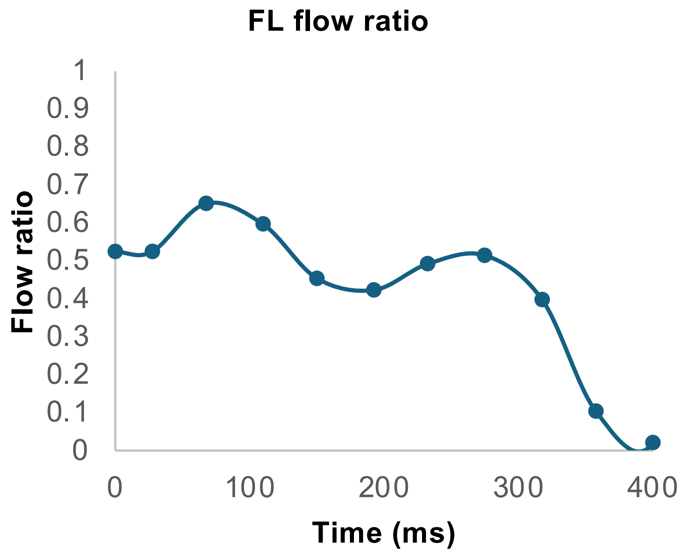

**Case 15** (End-systolic time = 360 ms) [Stable]

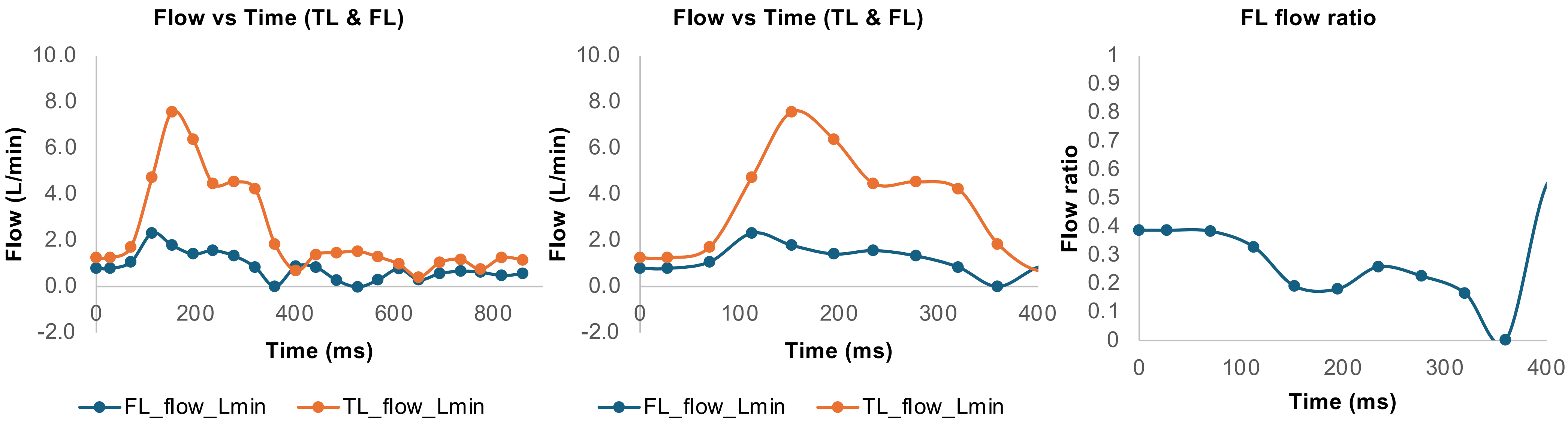


**Case 16** (End-systolic time = 373 ms) [Stable] + TEVAR

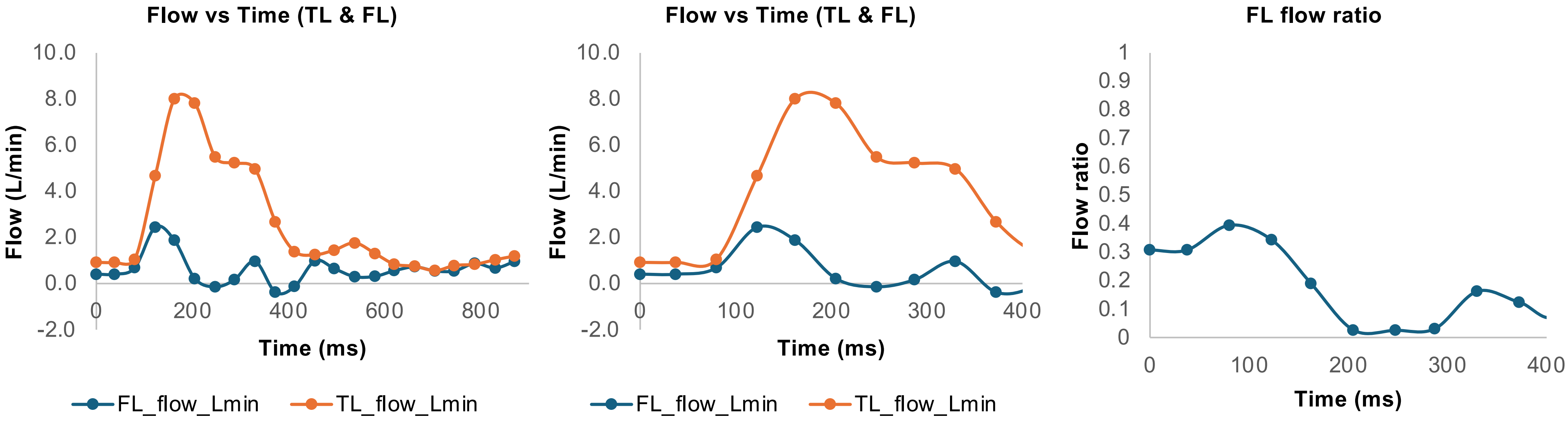

**Case 17** (End-systolic time = 328 ms) [Stable]

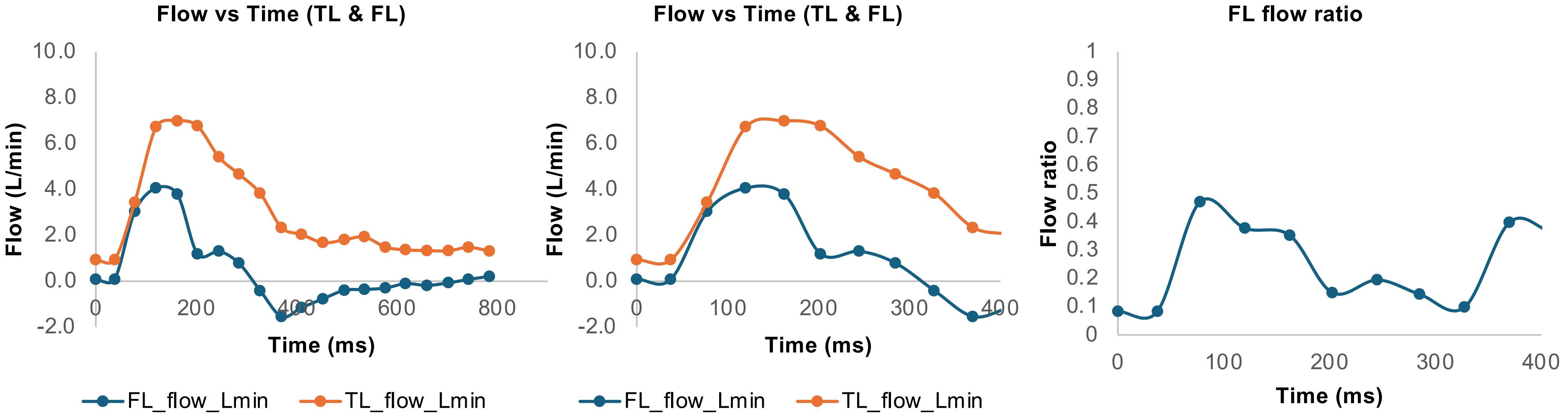


**Case 18** (End-systolic time = 383 ms) [Stable] + TEVAR

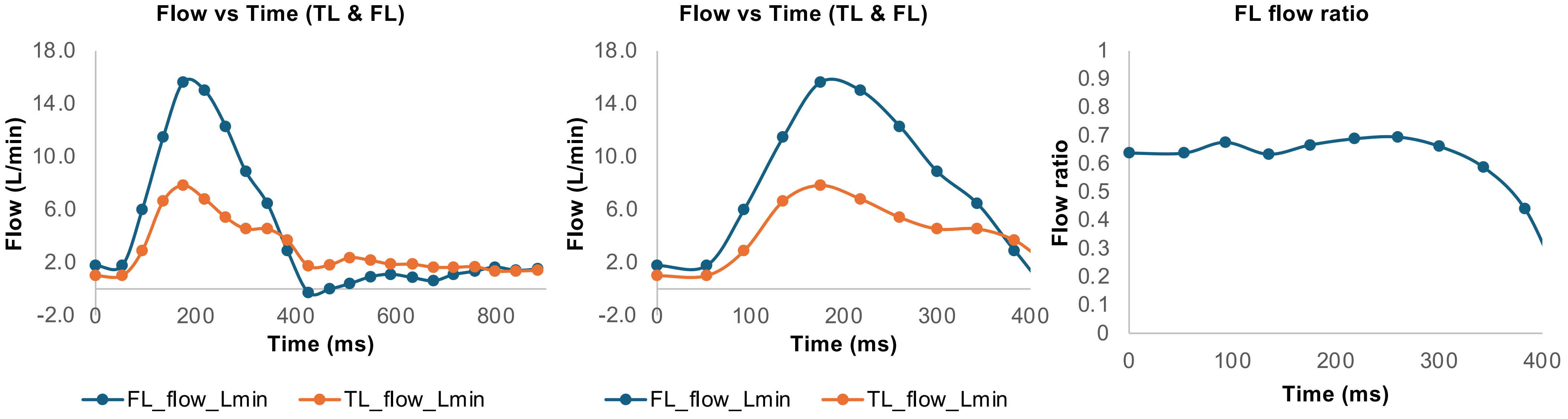

**Case 19** (End-systolic time = 338 ms) [Progression] + TEVAR

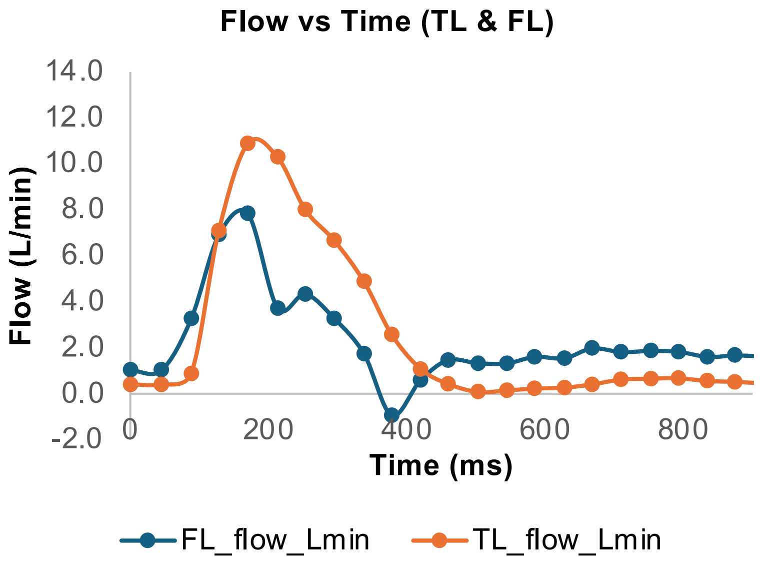


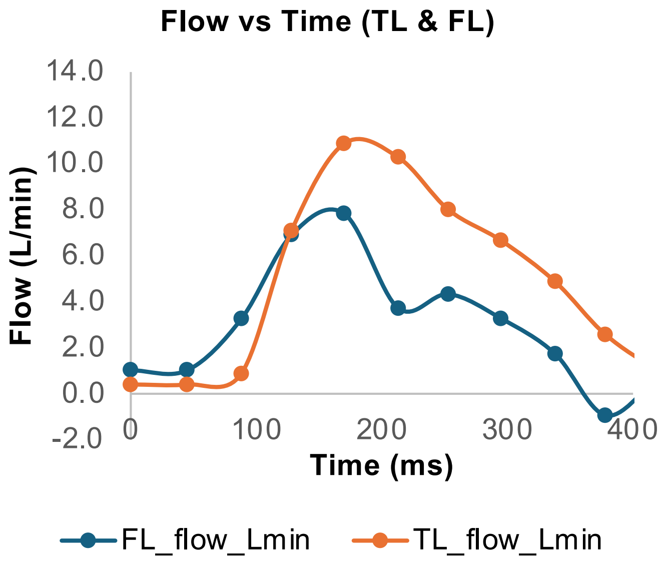


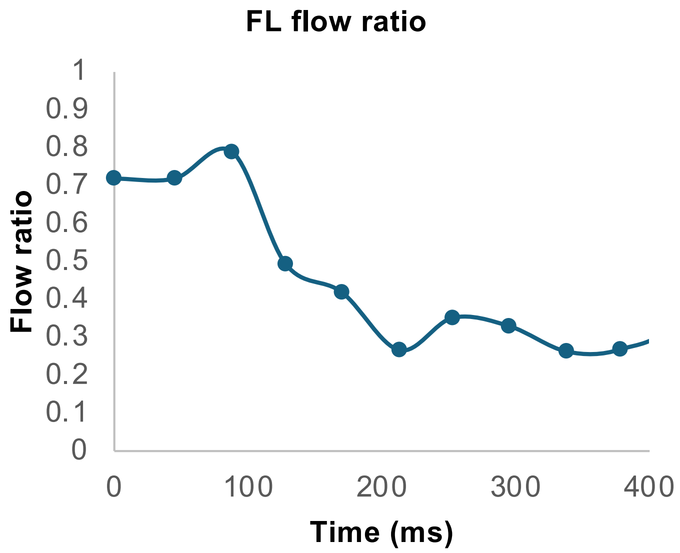


**Case 20** (End-systolic time = 345 ms) [Progression] + TEVAR

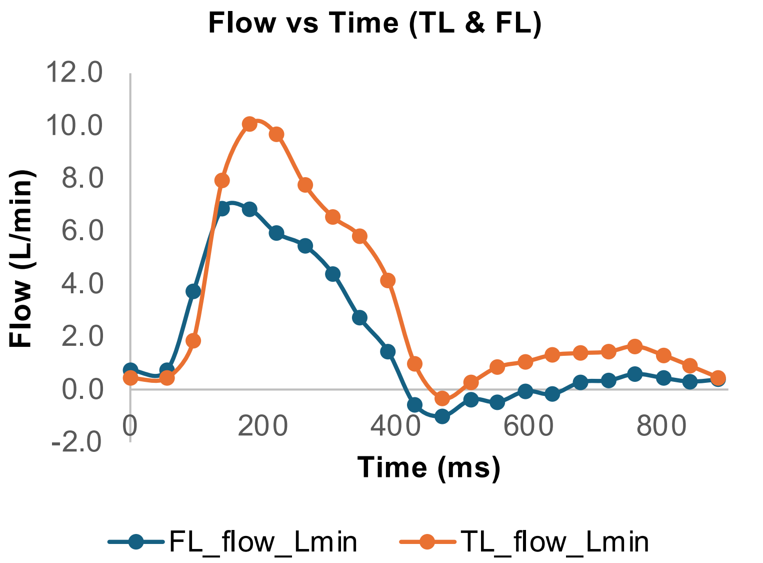


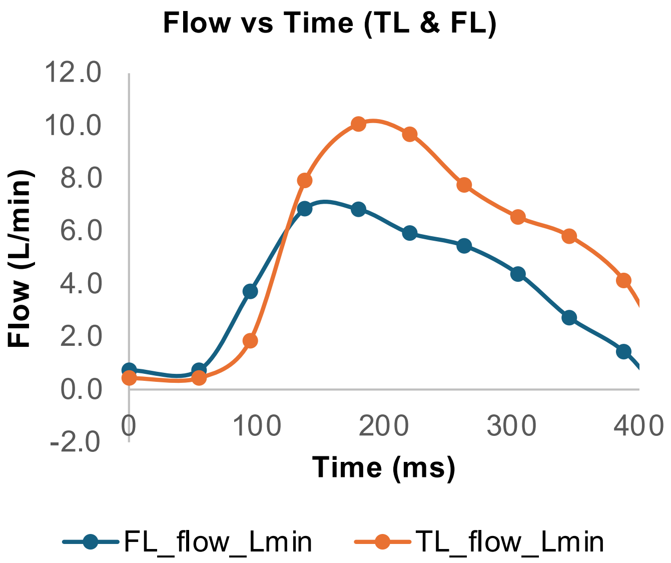


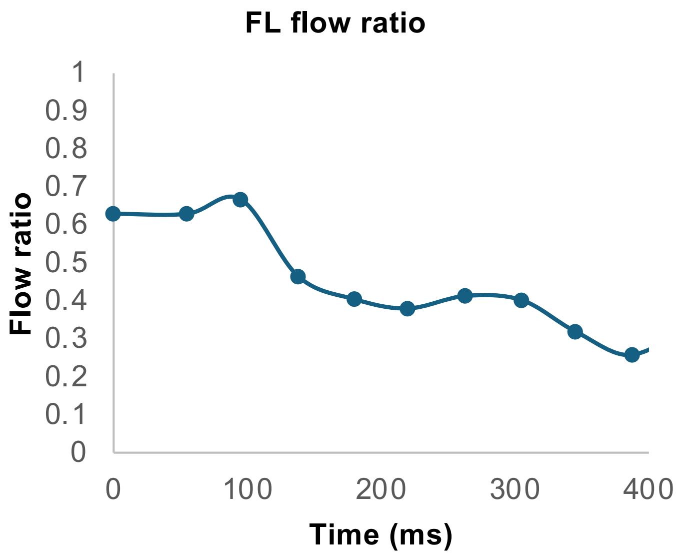

**Case 21** (End-systolic time = 263 ms) [Stable]

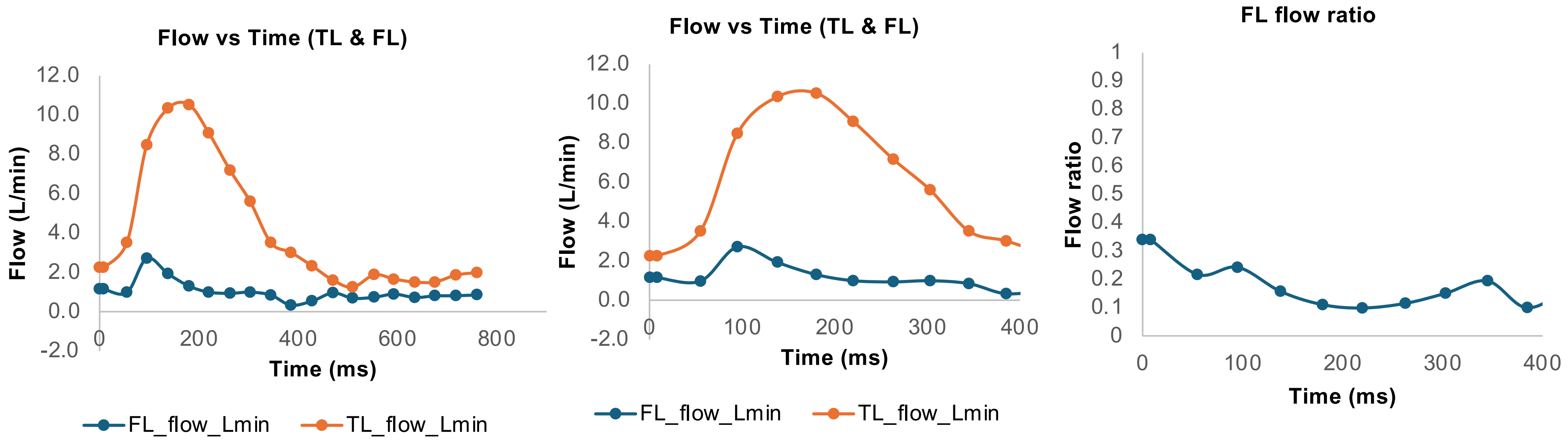


**Case 22** (End-systolic time = 335 ms) [Stable] + TEVAR

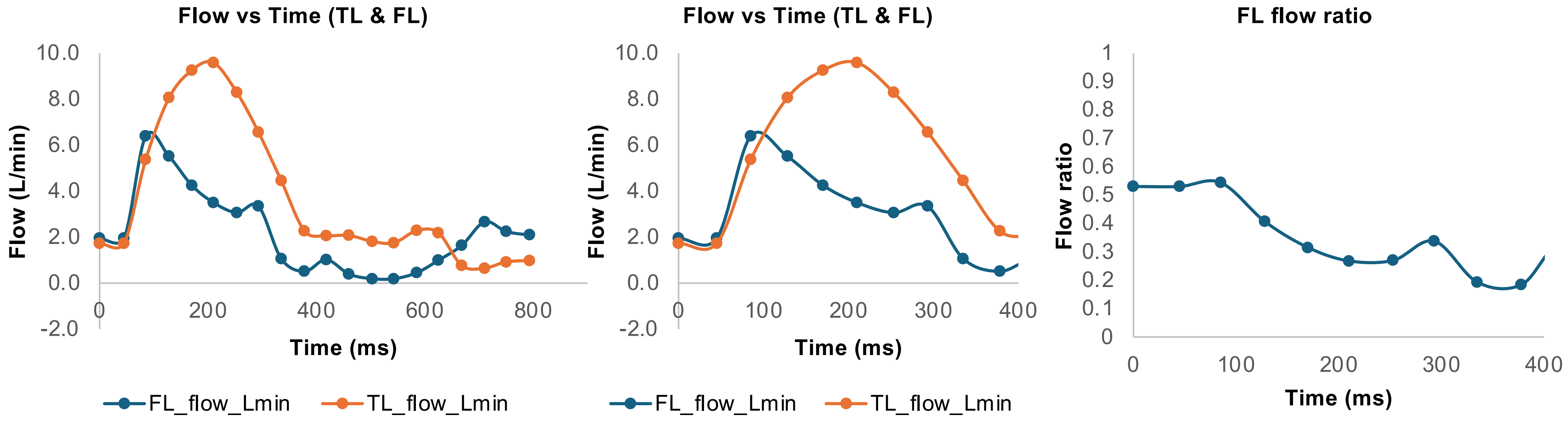

**Case 23** (End-systolic time = 345 ms) [Stable]

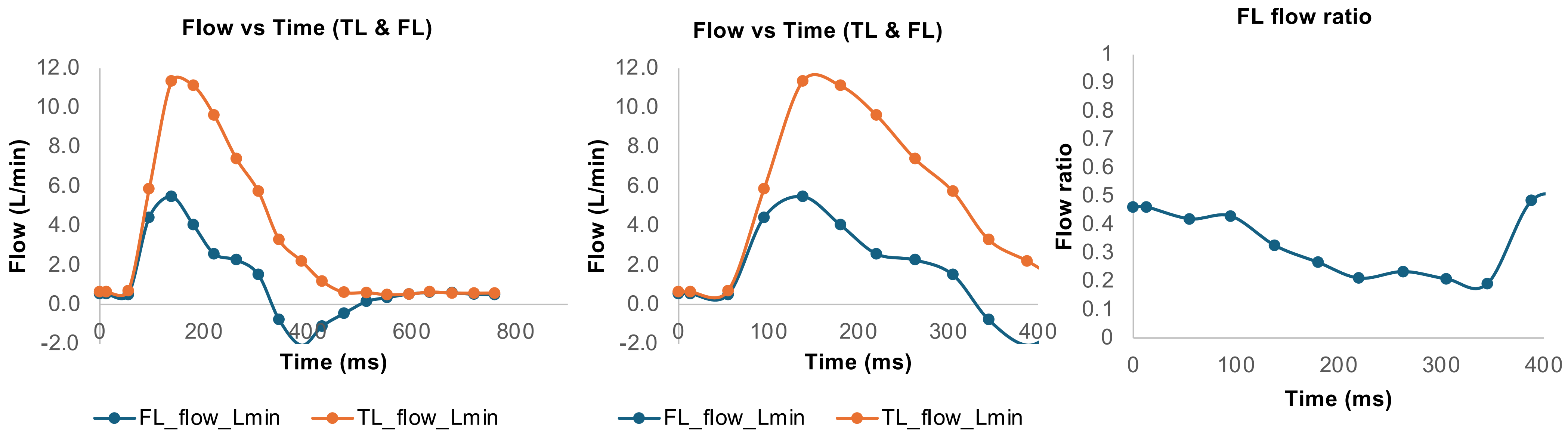


**Case 24** (End-systolic time = 243 ms) [Stable]

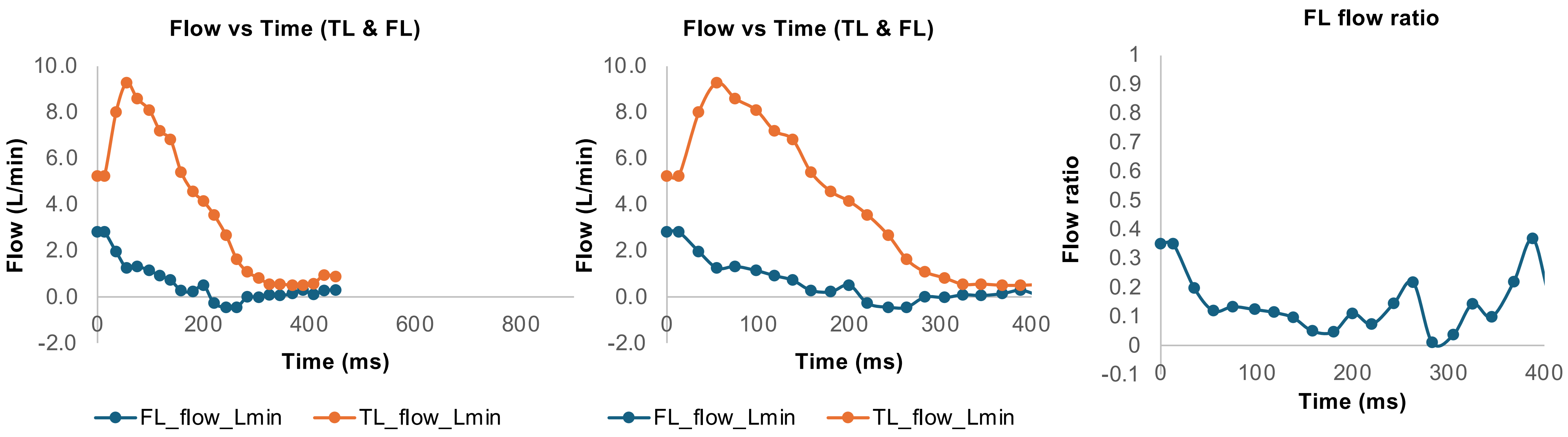

**Case 25** (End-systolic time = 440 ms) [Progression] + TEVAR

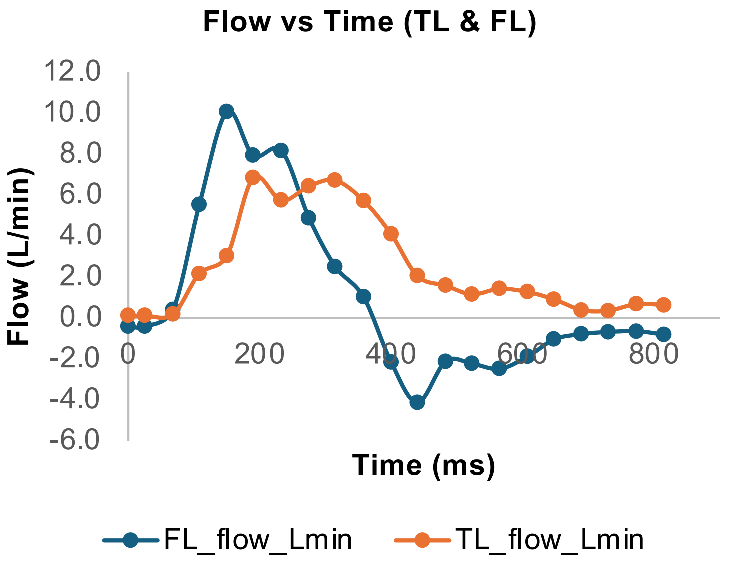


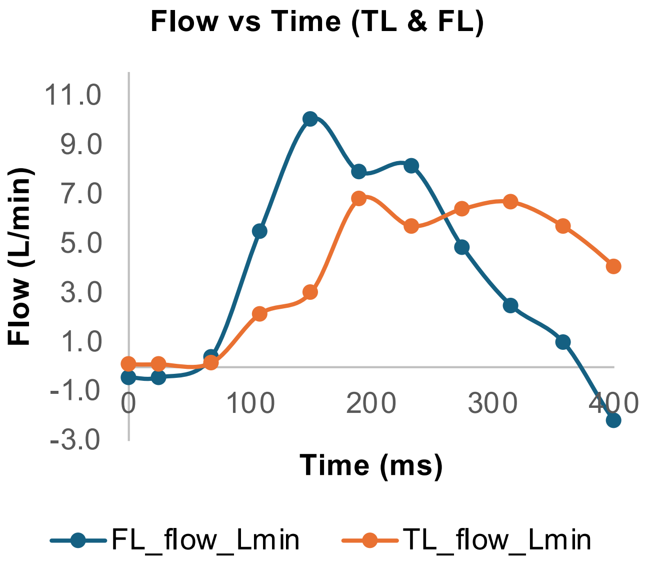


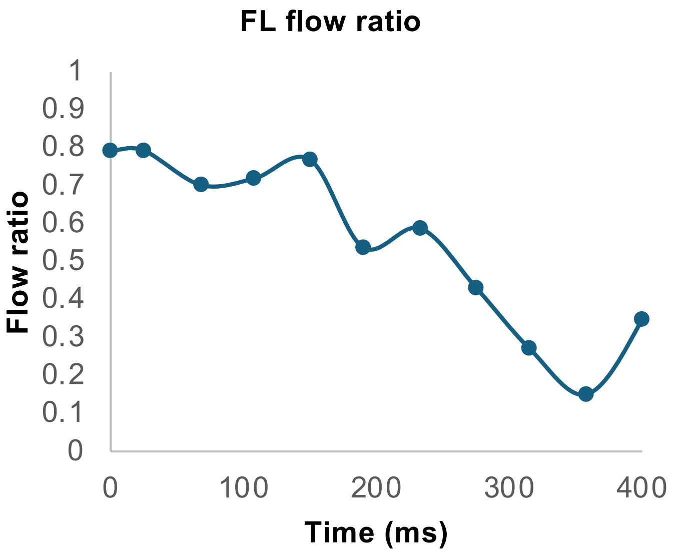


**Case 26** (End-systolic time = 330 ms) [Progression]

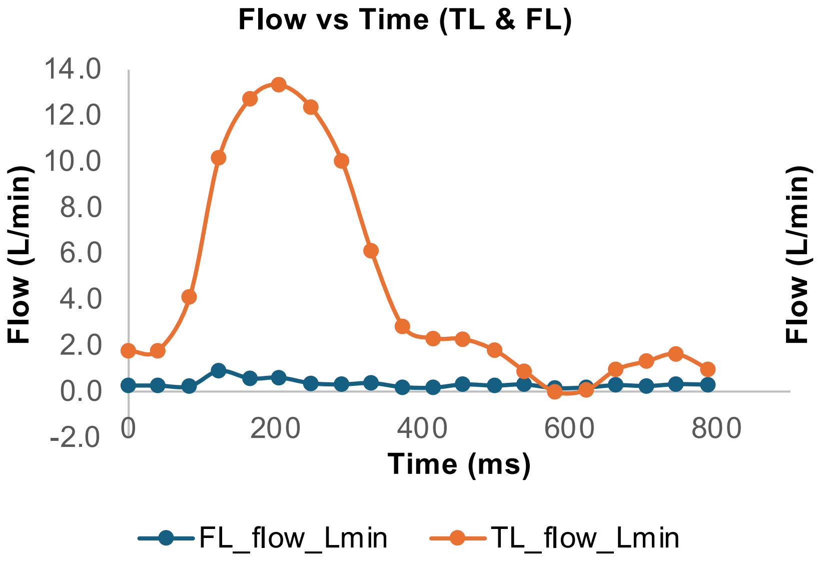


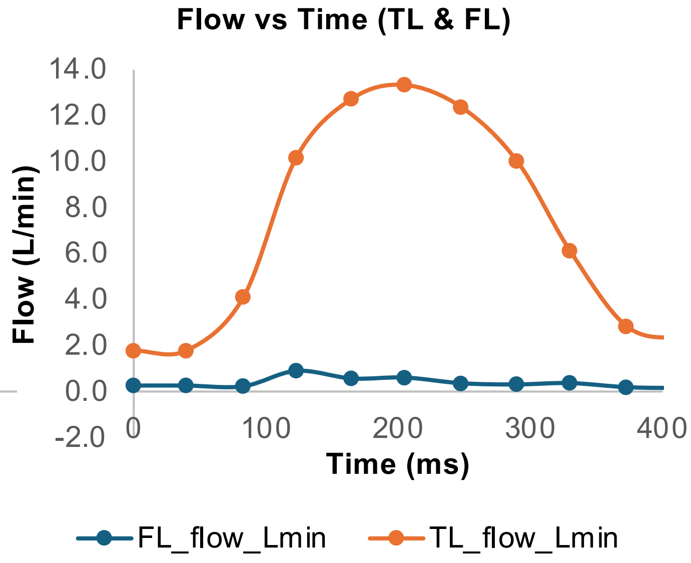


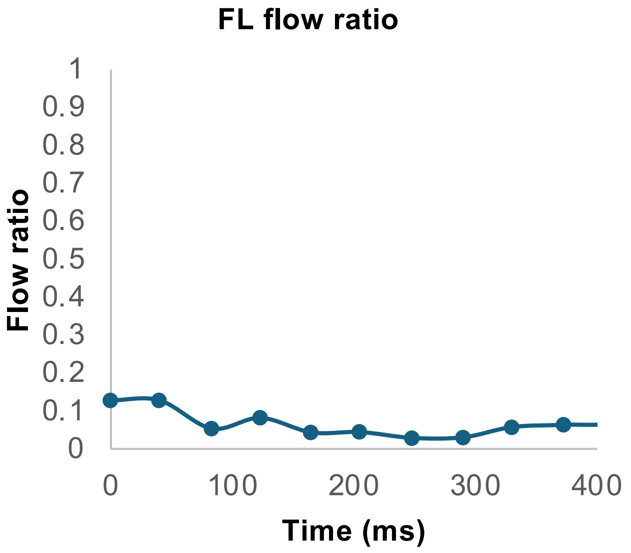

**Case 27** (End-systolic time = 325 ms) [Stable]

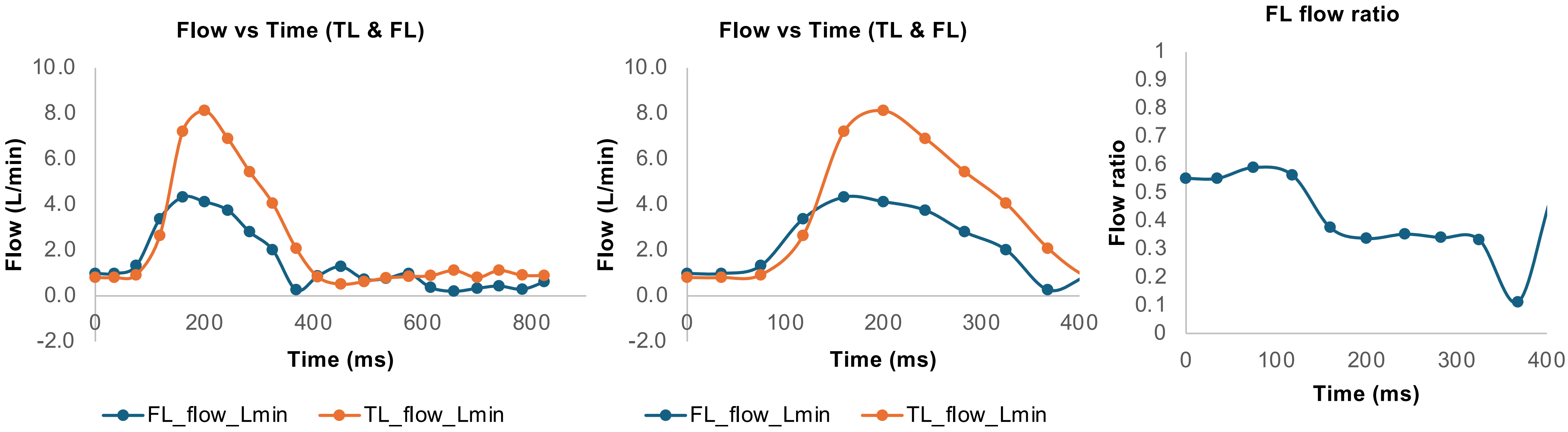


**Case 28** (End-systolic time = 330 ms) [Progression] + TEVAR

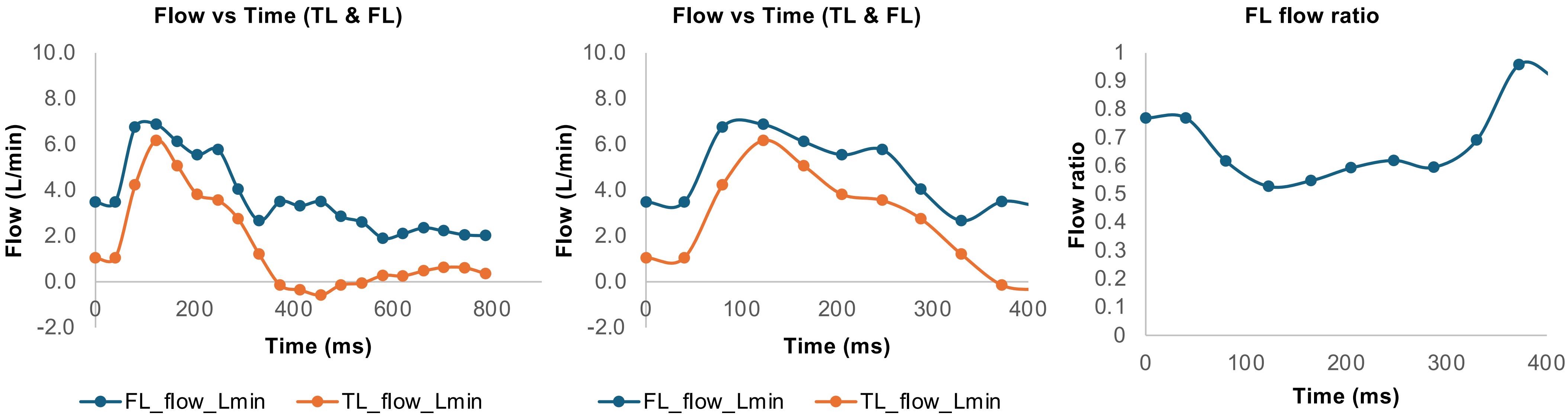

**Case 29** (End-systolic time = 388 ms) [Stable]

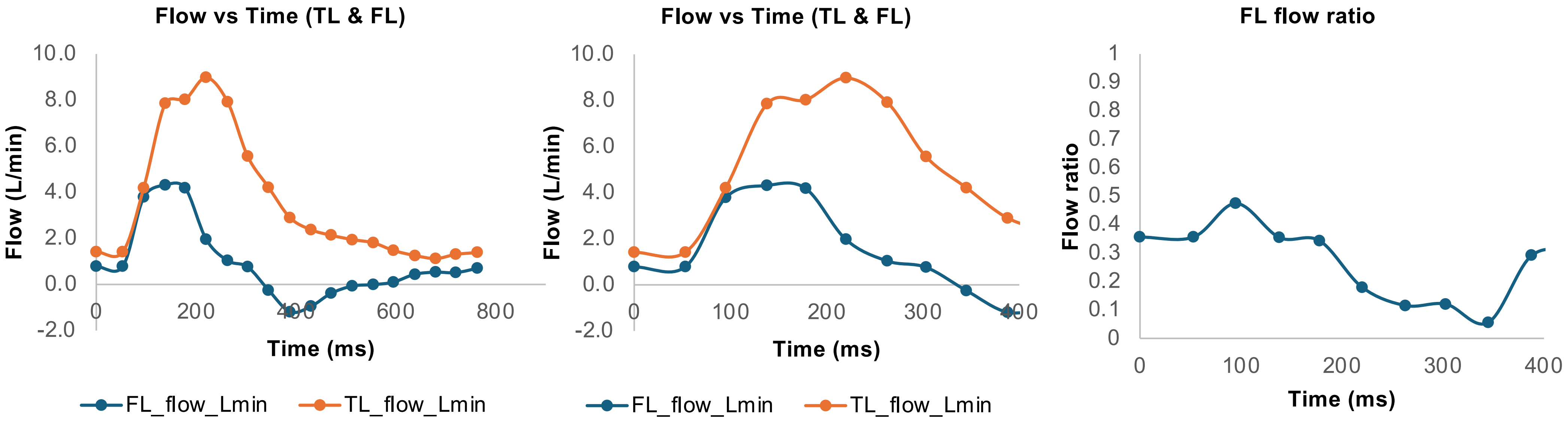


**Case 30** (End-systolic time = 325 ms) [Progression] + TEVAR

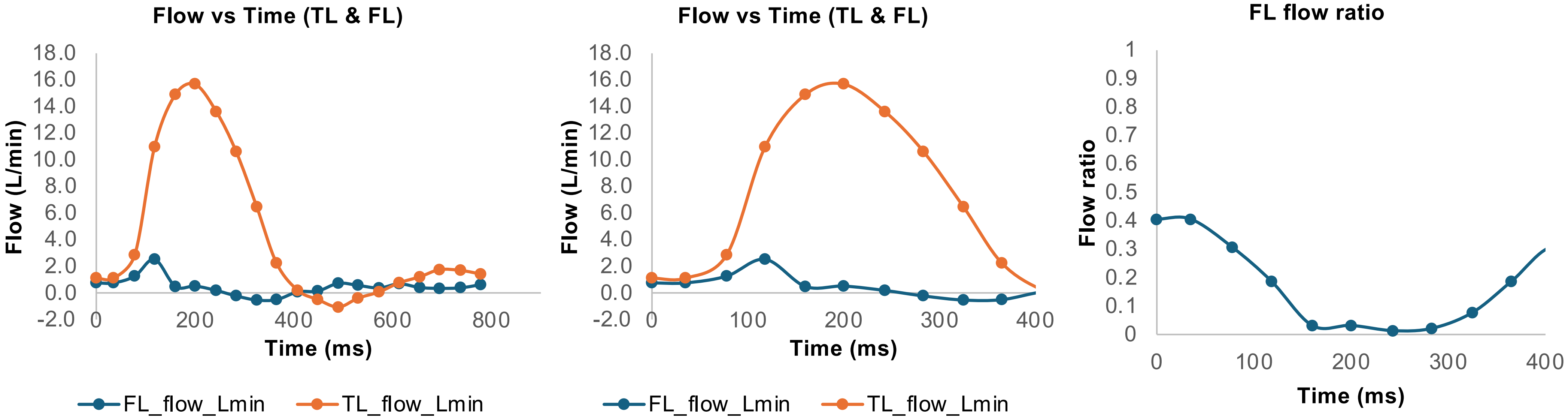

**Case 31** (End-systolic time = 385 ms) [Stable]

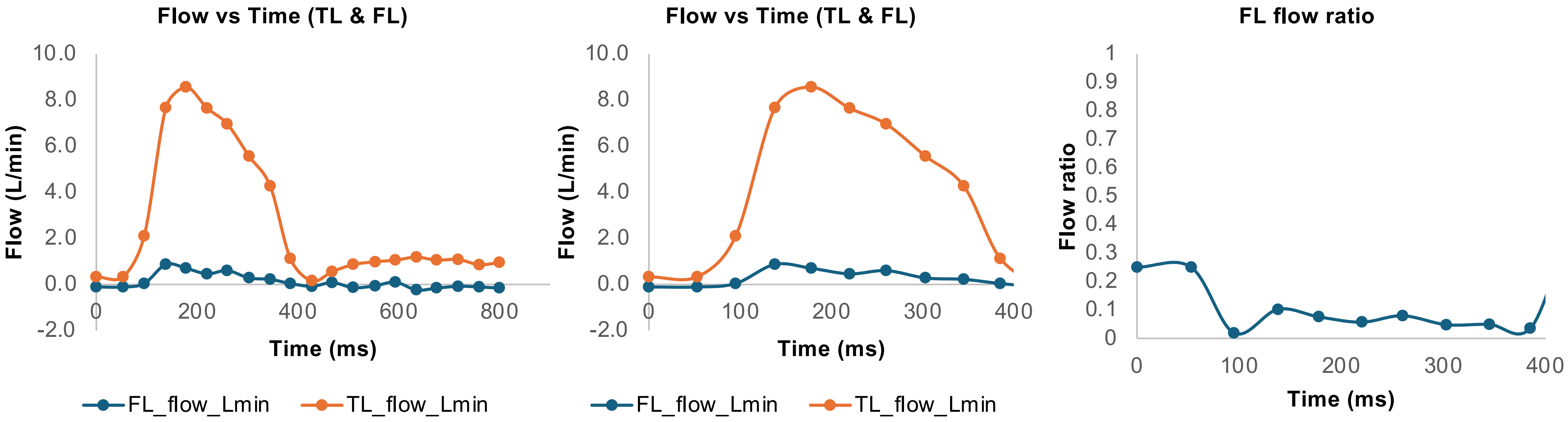


**Case 32** (End-systolic time = 328 ms) [Progression]

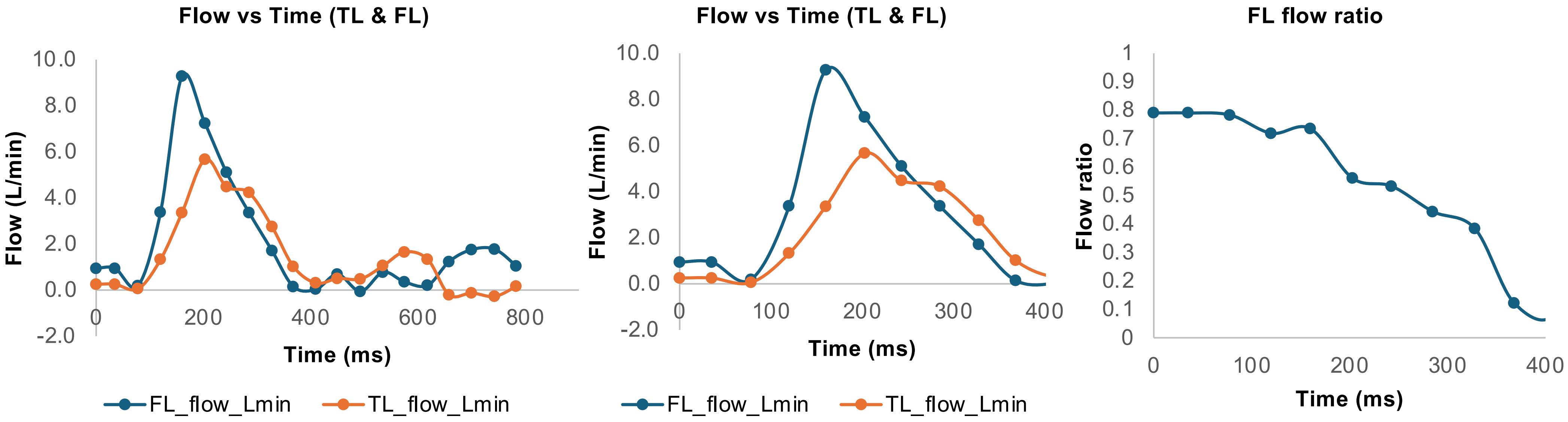

**Case 33** (End-systolic time = 358 ms) [Stable]

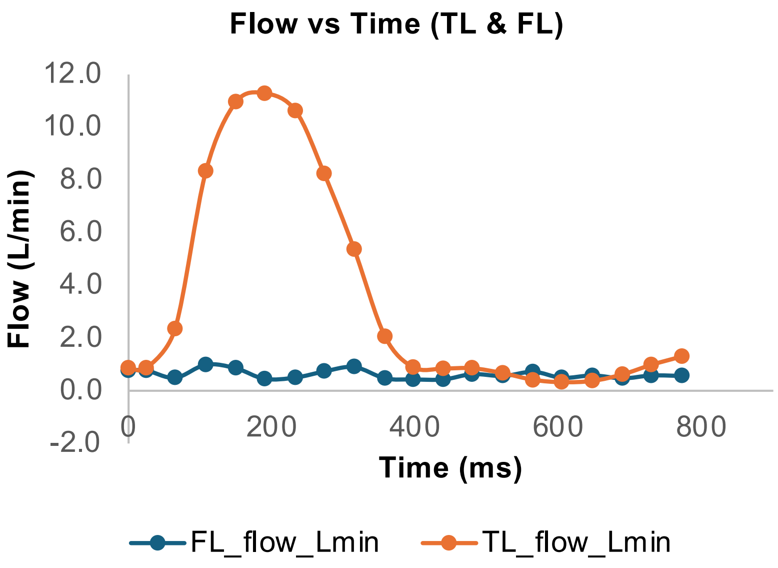


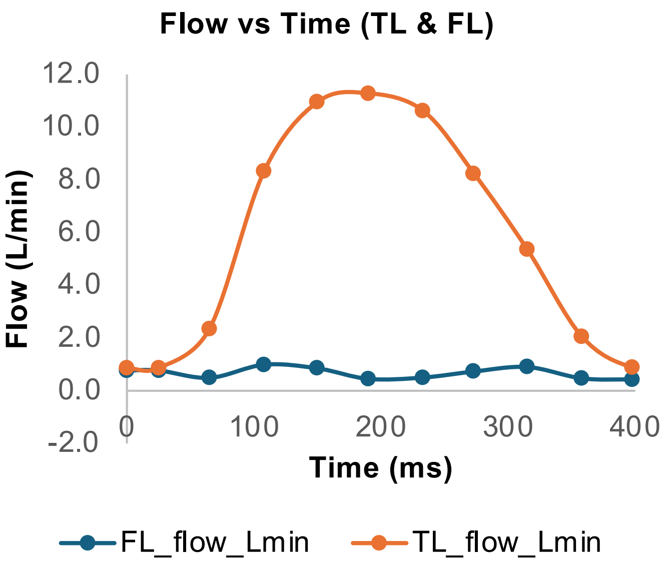


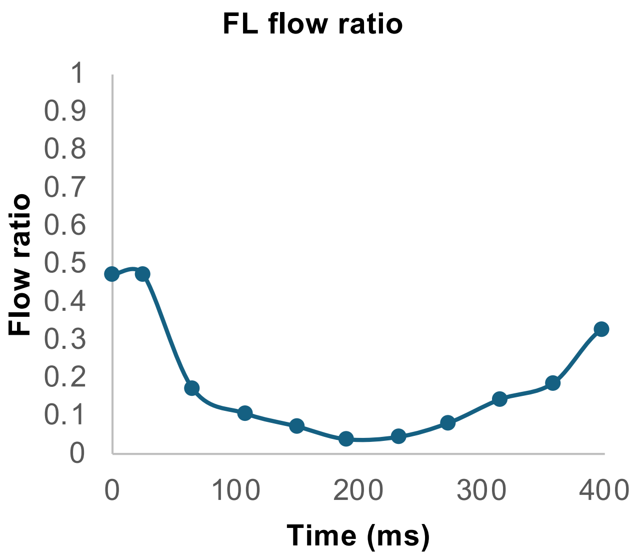


**Case 34** (End-systolic time = 342 ms) [Progression] + TEVAR

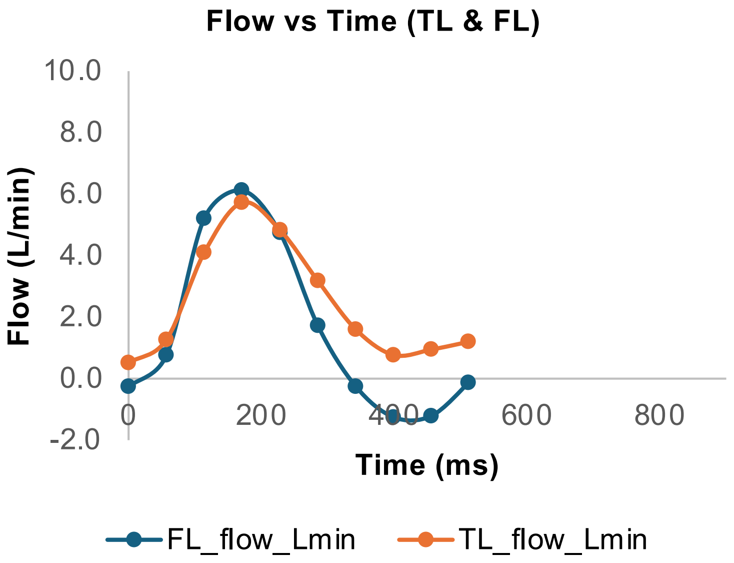


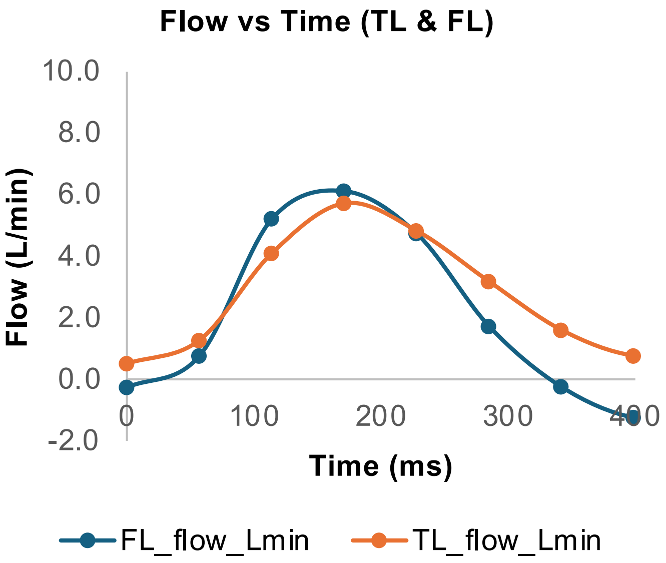


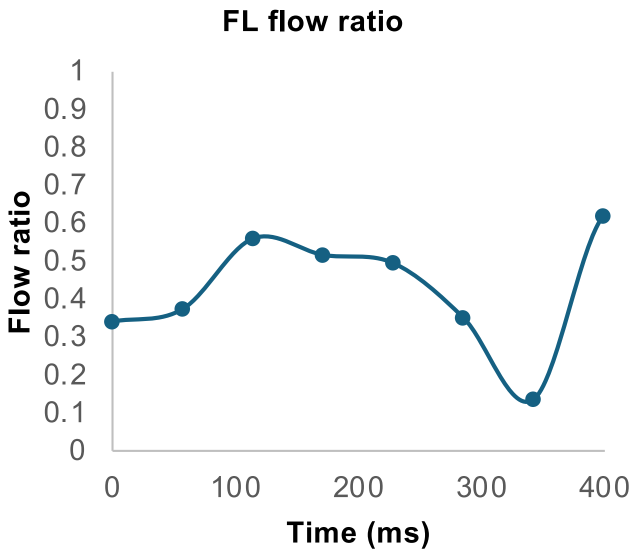

**Case 35** (End-systolic time = 323 ms) [Stable]

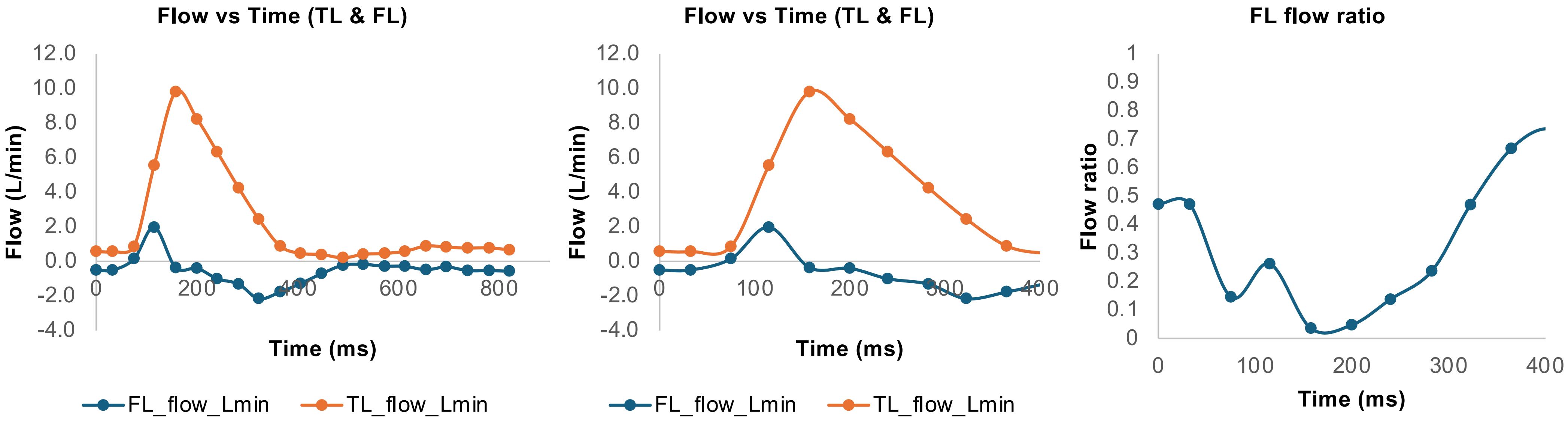


**Case 36** (End-systolic time = 333 ms) [Stable]

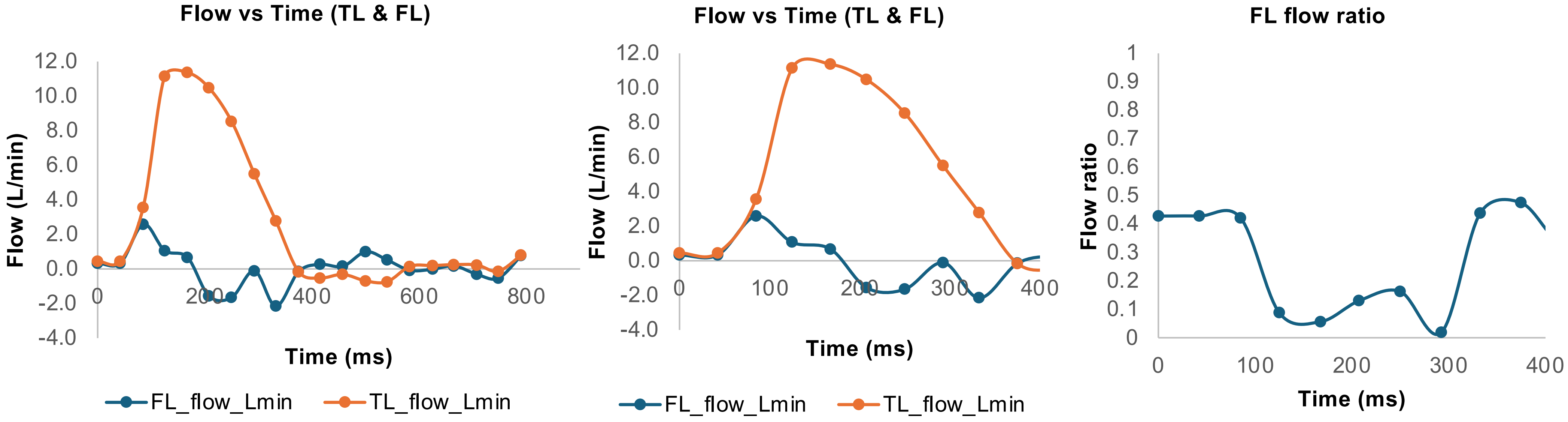

Supplement: ivag198_Supplementary_Data [file ivag198_supplementary_data.zip › Supplementary_Materials_M1-M2.pdf]
