# Supplementary material for: Haemodynamic Predictors of Early Aortic Growth in Uncomplicated Type B Dissection: A Cohort Study
Source: Interdiscip Cardiovasc Thorac Surg. 2026 Jul 8;41(7):ivag198. doi: 10.1093/icvts/ivag198 (PMC13408290; doi:10.1093/icvts/ivag198)
Supplement: ivag198_Supplementary_Data [file ivag198_supplementary_data.zip › Revised Supplementary Figures and Tables.docx]

**Supplementary Figures and Tables**


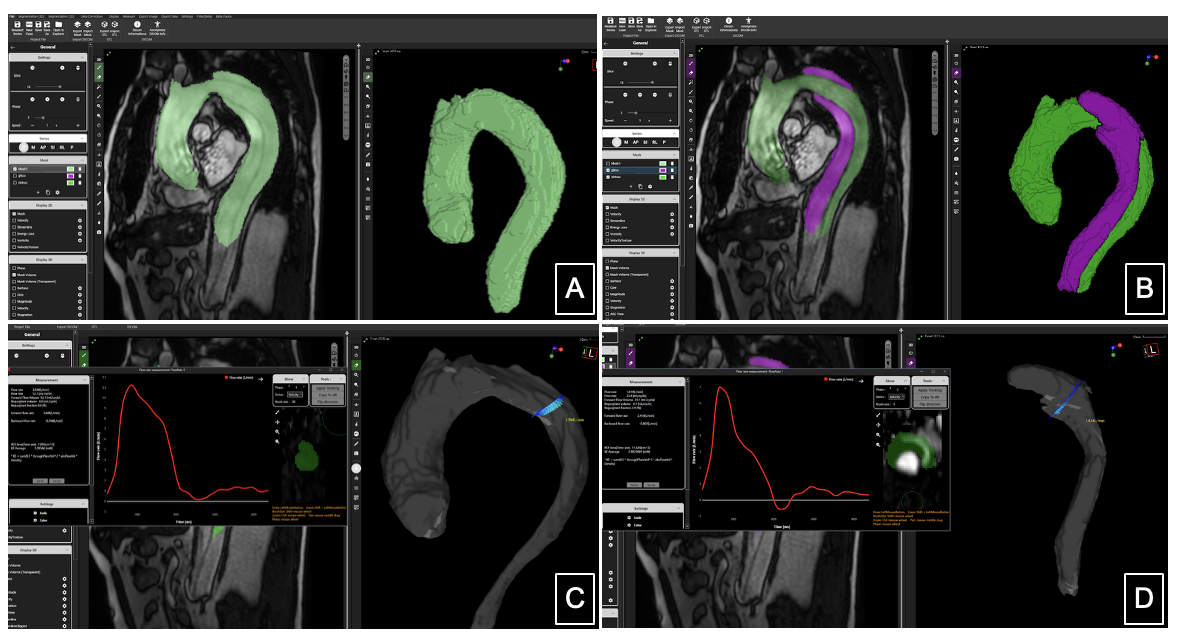


**Supplementary Figure S1. Workflow for quantification of true and false lumen flow using 4D flow MRI**

4D flow MRI is performed with the following imaging parameters: repetition time/echo time (TR/TE), 41.5/2.72 ms; flip angle, 8°; matrix size, 192 × 144 × 26; voxel size, 1.82 × 1.82 × 3.5 mm³, readout direction, sagittal. Velocity encoding is individually set to 1.5 m/s based on the peak blood flow velocity in the ascending aorta with an appropriate safety margin. The acquired DICOM datasets, including three-directional velocity fields, magnitude images, and cine position information, are imported into iTFlow (Version 3.4, Cardio Flow Design Inc., Tokyo, Japan). The thoracic aorta is segmented from the aortic valve to the descending aorta (A), after which masks of the true and false lumen are generated (B). A cross-sectional analysis plane is placed at the predefined level, allowing time-flow curves to be obtained for the true lumen (C) and false lumen (D), which are subsequently used for quantitative haemodynamic analysis.

4D flow MRI, four-dimensional flow magnetic resonance imaging

**Supplementary Figure S2. Representative true and false lumen flow waveforms and derived false lumen flow ratio**

TL, true lumen; FL, false lumen

**Supplementary Figure S3. Correlation between 4D flow MRI-derived flow parameters (TL gross flow and FL flow range) and early aortic growth rate in uTBAD**

(A, B) Scatter plots showing the association between the aortic growth rate, quantified as the ordinary least squares slope (mm/year) derived from serial CT measurements within the first year after onset, and the systolic TL gross flow (A) and FL flow range (B). Correlations are assessed using Spearman’s rank correlation across the full cohort (n = 36).

(C, D) Pearson’s correlation analysis for the same relationship after exclusion of statistical outliers (n = 30), defined using the interquartile range method.

Neither systolic TL gross flow nor FL flow range show a significant association with early aortic enlargement.

4D flow MRI, four-dimensional flow magnetic resonance imaging; uTBAD, uncomplicated type B aortic dissection; CT, computed tomography; TL, true lumen; FL, false lumen

**Supplementary Figure S4. Progression-free survival stratified by systolic false lumen flow index.** Kaplan–Meier curves for freedom from CT-defined aortic progression within the first year, comparing patients with a high (above the cohort median) versus low (at or below the median) systolic FL flow index. Shaded areas represent 95% confidence intervals, and the number of patients at risk is shown below the horizontal axis. The between-group difference was assessed using the log-rank test. Dichotomisation at the median was performed for illustration only; all inferential analyses (Cox regression) treated the FL flow index as a continuous variable. CT, computed tomography; FL, false lumen

**Supplementary Equations**

1. FL flow index

$$FL flow ratio= R\left( t \right)=\frac{\left| Q_{FL}\left( t \right) \right|}{\left| Q_{FL}\left( t \right) \right|+\left| Q_{TL}\left( t \right) \right|}\text{,}$$

$$\text{FL flow index}=\frac{1}{T_{sys}}\int_{ADDIN EN.CITE <EndNote><Cite ExcludeYear="1"><Author>Charles L. McIntosh</Author><Year>1992</Year><RecNum>1919</RecNum><record><rec-number>1919</rec-number><foreign-keys><key app="EN" db-id="r5wfzwz5tpt9sbexazovdzxxf0ttwr9exaee" timestamp="1682990930" guid="393172f9-0ec0-49db-bcd5-175eda798b46">1919</key></foreign-keys><ref-type name="Journal Article">17</ref-type><contributors><authors><author>Charles L. McIntosh, MD, PhD, Barry J. Maron, MD, </author><author>Richard 0. Cannon III, MD, and Heinrich G. Klues, MD</author></authors></contributors><titles><title>Initial Results of Combined Anterior Mitral\&\#xD;Leaflet Plication and Ventricular Septal\&\#xD;Myotomy-Myectomy for Relief of Left\&\#xD;Ventricular Outflow Tract\&\#xD;Obstruction in Patients With\&\#xD;Hypertrophic Cardiomyopathy</title><secondary-title>Circulation</secondary-title></titles><periodical><full-title>Circulation</full-title></periodical><pages>60-67</pages><volume>86</volume><num-vols>Ⅱ</num-vols><dates><year>1992</year></dates><urls></urls></record></Cite></EndNote>0}^{T_{sys}} R\left( t \right) dt$$

Definition: The FL flow index is defined as the systolic time-averaged ratio of the instantaneous FL flow ratio, calculated as the proportion of FL flow to the total aortic flow (FL + TL), representing the relative contribution of the FL.

2. FL gross flow (L/min)

$$\text{FL gross flow}=\frac{1}{T_{sys}}\int_{0}^{T_{sys}} \left| Q_{FL}\left( t \right) \right| dt$$

Definition: The average absolute FL flow during systole is expressed as the time-normalised mean flow rate*.*

3. TL gross flow (L/min)

$$\text{TL gross flow}=\frac{1}{T_{sys}}\int_{0}^{T_{sys}} \left| Q_{TL}\left( t \right) \right| dt$$

Definition: The average absolute true lumen flow during systole is expressed as the time-normalised mean flow rate.

4. FL flow range (L/min)

$$FL flow range=max\left( Q_{FL}\left( t \right) \right)-min\left( Q_{FL}\left( t \right) \right)$$

Definition: The difference between the maximum and minimum instantaneous false lumen flows during systole reflects the pulsatility amplitude.

5. Aortic growth rate (OLS slope, mm/year)

$$b=\frac{\sum_{i=1}^{n} \left( t_{i}-\bar{t} \right)\left( D_{i}-\bar{D} \right)}{\sum_{i=1}^{n} \left( t_{i}-\bar{t} \right)^{2}}$$

Definition: Slope (b) is derived from the OLS linear regression of serial aortic diameter measurements (D_i_) against observation time (t_i_), representing the annualised rate of aortic enlargement.

FL, false lumen; TL, true lumen; OLS, ordinary least squares; *T*_sys_, end-systolic time.

$Q_{\mathrm{FL}}\left( t \right)$ and $Q_{\mathrm{TL}}\left( t \right)$ represent the instantaneous flow rates in the FL and TL.

**Supplementary Table S1. Classification of CT-based aortic progression status and TEVAR in patients with uTBAD**

| Classification | Number of patients | TEVAR performed, n (%) |
| --- | --- | --- |
| Progression | 16 | 12 (75) |
| Criterion A only | 2 | 2 (100) |
| Criterion B only | 10 | 6 (60) |
| Criteria A and B | 4 | 4 (100) |
| Stable | 20 | 3 (15) |

CT, computed tomography; TEVAR, thoracic endovascular aortic repair; uTBAD, uncomplicated type B aortic dissection

**Supplementary Table S2. Sensitivity analysis comparing systolic 4D flow MRI-derived haemodynamic parameters after reclassification of patients undergoing TEVAR into the progression group**

| Variable | Progression + TEVAR  (n = 19) | Stable  (n = 17) | P value |
| --- | --- | --- | --- |
| CT measurement |  |  |  |
| MAD onset, mm | 37.3 [32.2, 39.2] | 31.9 [30.9, 34.8] | 0.01 |
| MAD latest follow-up, mm | 43.3 [40.3, 47.8] | 36.7 [34.2, 38.4] | < 0.01 |
| Aortic growth rate, mm/year | 12.2 [9.1, 55.8] | 4.2 [2.8, 5.6] | < 0.01 |
| Entry size, mm | 9.0 [6.1, 11.3] | 7.8 [6.9, 10.0] | 0.79 |
| MAD onset ≥ 40 mm, n (%) | 5 (26.3) | 1 (5.9) | 0.18 |
| Entry size > 10 mm, n/ N (%) | 8/19 (42.1) | 4/13 (30.8) | 0.71 |
| Partial thrombosis, n (%) | 7 (36.8) | 8 (47.1) | 0.53 |
| FL at lesser curvature, n (%) | 1 (5.3) | 3 (17.6) | 0.33 |
| FL > 22 mm at onset, n (%) | 5 (26.3) | 1 (5.9) | 0.18 |
| 4D flow MRI parameters |  |  |  |
| FL flow index | 0.49 [0.24, 0.62] | 0.29 [0.20, 0.35] | 0.02 |
| FL gross flow, L/min | 3.78 [1.53, 4.32] | 1.62 [1.10, 2.58] | 0.01 |
| TL gross flow, L/min | 4.86 [3.93, 7.29] | 5.26 [4.78, 6.85] | 0.55 |
| FL flow range, L/min | 6.01 [3.34, 6.97] | 4.14 [2.31, 5.50] | 0.08 |

Values are presented as median [interquartile range] or n (%). Entry size and entry size >10 mm are unavailable in four patients because the primary entry tear could not be identified on CT.

CT, computed tomography; MAD, maximum aortic diameter; 4D flow MRI, four-dimensional flow magnetic resonance imaging; FL, false lumen; TL, true lumen

**Supplementary Table S3. Multivariable sensitivity analysis after reclassification of patients undergoing TEVAR into the progression group**

| Variable | OR | 95% CI | p value |
| --- | --- | --- | --- |
| FL flow index (per 1-SD increase) | 3.31 | 1.16–9.45 | 0.025 |
| Baseline MAD (per 1-SD increase) | 4.51 | 1.28–15.9 | 0.019 |

Multivariable logistic regression with the progression-plus-TEVAR group (n = 19) versus the stable group (n = 17) as the dependent variable. Predictors were standardised (each expressed per 1-SD increase). Events-per-variable ratio ≈ 9.5. McFadden pseudo-R^2^ = 0.33.

CI, confidence interval; FL, false lumen; MAD, maximum aortic diameter; OR, odds ratio; SD, standard deviation

**Supplementary Table S4. Exploratory Cox proportional hazards analysis for time to CT-based aortic progression**

| Variable | HR | 95% CI | p value |
| --- | --- | --- | --- |
| FL flow index (per 1-SD increase) | 2.21 | 1.33–3.67 | 0.002 |
| Baseline MAD (per 1-SD increase) | 1.62 | 1.03–2.56 | 0.038 |

Cox proportional hazards regression for time to CT-based aortic progression; observation was censored at TEVAR or at 12 months, whichever occurred first. Predictors were standardised (each expressed per 1-SD increase). Sixteen events occurred among 36 patients (events-per-variable ratio ≈ 8). The proportional hazards assumption was satisfied (Schoenfeld test, p ≥ 0.23 for both covariates), and Harrell’s concordance index was 0.78.

CI, confidence interval; CT, computed tomography; FL, false lumen; HR, hazard ratio; MAD, maximum aortic diameter; SD, standard deviation; TEVAR, thoracic endovascular aortic repair
